# Zyxin Links Fat Signaling to the Hippo Pathway

**DOI:** 10.1371/journal.pbio.1000624

**Published:** 2011-06-07

**Authors:** Cordelia Rauskolb, Guohui Pan, B. V. V. G. Reddy, Hyangyee Oh, Kenneth D. Irvine

**Affiliations:** Howard Hughes Medical Institute, Waksman Institute, and Department of Molecular Biology and Biochemistry, Rutgers, The State University of New Jersey, Piscataway, New Jersey, United States of America; University of Zurich, Switzerland

## Abstract

The Hippo signaling pathway has a conserved role in growth control and is of fundamental importance during both normal development and oncogenesis. Despite rapid progress in recent years, key steps in the pathway remain poorly understood, in part due to the incomplete identification of components. Through a genetic screen, we identified the *Drosophila* Zyxin family gene, *Zyx102* (Zyx), as a component of the Hippo pathway. Zyx positively regulates the Hippo pathway transcriptional co-activator Yorkie, as its loss reduces Yorkie activity and organ growth. Through epistasis tests, we position the requirement for Zyx within the Fat branch of Hippo signaling, downstream of Fat and Dco, and upstream of the Yorkie kinase Warts, and we find that Zyx is required for the influence of Fat on Warts protein levels. Zyx localizes to the sub-apical membrane, with distinctive peaks of accumulation at intercellular vertices. This partially overlaps the membrane localization of the myosin Dachs, which has similar effects on Fat-Hippo signaling. Co-immunoprecipitation experiments show that Zyx can bind to Dachs and that Dachs stimulates binding of Zyx to Warts. We also extend characterization of the Ajuba LIM protein Jub and determine that although Jub and Zyx share C-terminal LIM domains, they regulate Hippo signaling in distinct ways. Our results identify a role for Zyx in the Hippo pathway and suggest a mechanism for the role of Dachs: because Fat regulates the localization of Dachs to the membrane, where it can overlap with Zyx, we propose that the regulated localization of Dachs influences downstream signaling by modulating Zyx-Warts binding. Mammalian Zyxin proteins have been implicated in linking effects of mechanical strain to cell behavior. Our identification of Zyx as a regulator of Hippo signaling thus also raises the possibility that mechanical strain could be linked to the regulation of gene expression and growth through Hippo signaling.

## Abstract 2

Using genetic and molecular analyses, the authors identify Zyx as a positive regulator of Hippo signaling and characterize its role within the pathway.

## Author Summary

Processes that control cell numbers are essential during normal development, when they are required to generate organs of the correct size, and during cancinogenesis, when they influence tumor growth. The Hippo pathway is an intercellular signaling pathway that relays information about cell-cell contact and cell polarity to a signal transduction pathway that regulates the transcription of genes controlling cell numbers. The role of Hippo signaling in controlling growth is conserved from fruit flies to humans, but many aspects of the Hippo signal transduction pathway remain poorly understood. In this article, we identify Zyx as a previously unknown component of the Hippo pathway in *Drosophila*, and characterize its role within the pathway. We show that Zyx plays an essential role in a branch of Hippo signaling that involves the transmembrane receptor protein Fat and its target Dachs, which is a myosin family protein. Our results suggest a model in which Fat regulates the localization of Dachs, Dachs subsequently binds Zyx, stimulating its binding with the kinase Warts/Lats, and thereby regulates downstream signaling events. Zyx is conserved in vertebrates and we suggest that vertebrate Zyx proteins might also be involved in the regulation of Hippo signaling and, thereby, organ growth.

## Introduction

The Hippo pathway has emerged as an important regulator of growth during metazoan development, and its dysregulation is implicated in diverse cancers [1]–[3]. Hippo signaling is effected by transcriptional co-activator proteins, Yorkie (Yki) in *Drosophila* and YAP and TAZ in mammals [4]. Three interconnected, upstream branches of Hippo signaling have been characterized in *Drosophila*: Fat-dependent, Expanded-dependent, and Merlin-dependent [1]–[3]. These upstream branches converge on the kinase Warts (Wts), which can phosphorylate Yki. Phosphorylated Yki is retained in the cytoplasm, whereas unphosphorylated Yki can enter the nucleus and, in conjunction with DNA-binding partners, promote the transcription of downstream genes. Upstream branches of Hippo signaling regulate both the activity of Wts and its abundance. Our understanding of many steps in Hippo signaling remains fragmentary, in part due to incomplete identification of pathway components. Here, we describe the identification of Zyx102 (Zyx, FBgn0011642) as a novel component of Hippo signaling and characterize its role in the pathway.

Fat is large cadherin that acts as a transmembrane receptor for one branch of Hippo signaling [1]–[3],[5]. Fat-Hippo signaling influences the levels of Wts protein [6]. The molecular mechanism by which this is achieved is not understood, but *dachs* is genetically required for the influence of Fat on Wts levels, downstream gene expression, and organ growth [6]–[8]. Fat regulates the localization of Dachs to the sub-apical membrane: when *fat* is mutant, Dachs accumulates on the membrane around the entire circumference of the cell, and when Fat is over-expressed, Dachs is mostly cytoplasmic [7]. In imaginal discs and optic neuroepithelia, Dachs membrane localization is polarized within the plane of the tissue; this polarization reflects the graded expression of the Fat ligand Dachsous and the Fat pathway modulator Four-jointed [7],[9],[10]. The correlation of Dachs localization with Fat activity implicates Dachs regulation as a key step in Fat signaling, but how Dachs localization influences downstream events is unknown.

Zyx is a *Drosophila* homologue of the vertebrate Zyxin, Lipoma preferred partner (LPP), and Thyroid-receptor interacting protein 6 (TRIP6) proteins [11],[12]. These proteins have three conserved LIM domains at their C-terminus, and they have been implicated in both cytoskeletal and transcriptional regulation [13]–[15]. Gene-targeted mutations in murine *Zyxin* or *Lpp* have no significant effect on mouse development, presumably due to redundancy among family members [16],[17]. Translocations involving *LPP* identified it as an oncogene involved in lipomas and other cancers [13]. In cultured cell assays, Zyxin and its paralogues can affect cell motility and actin polymerization and can localize to focal adhesions and adherens junctions [13],[15],[18]. Notably, Zyxin has been implicated as playing a key role in mechanotransduction, as its localization to focal adhesions can be influenced by the application of mechanical tension to cells in culture [18].

We report here that Zyx is an essential component of the Fat-Hippo signaling pathway, required for normal Yki activity and growth in *Drosophila*. Using genetic epistasis tests, we position the requirement for *Zyx* in between *fat* and *wts*. Binding studies show that Zyx protein binds to Dachs and binds to Wts in a Dachs-regulated manner. Our observations suggest a model in which the regulated localization of Dachs to the membrane regulates Zyx-Wts binding, which then promotes Wts degradation. Dachs is a myosin protein, and its myosin motor domain contributes to interactions with Zyx and Wts, which raises the possibility that additional myosins might regulate Zyx-Wts interactions in other contexts.

## Results

In a screen for additional components of the Fat and Hippo pathways, we examined a collection of transgenic flies expressing UAS-hairpin constructs, which mediate RNAi. We focused on the X and 4^th^ chromosomes, which are under-represented in traditional genetic screens, and looked for phenotypes when these RNAi lines were expressed in the notum under *pnr-Gal4* control, and in the wing under *vg-Gal4* control. To enhance the strength of RNAi, the screening was done in flies expressing Dicer2 from a *UAS-dcr2* transgene [19]. One hundred and forty-eight lines exhibiting either altered tissue growth or lethality were then re-screened for possible effects on Fat-Hippo signaling by assaying the expression of downstream targets of the pathway, Wingless (Wg) and *thread* (*th*, more commonly referred to as *Diap1*) [20],[21], in wing discs in which RNAi lines were expressed in anterior cells under *ci-Gal4* control (Table S1). The most promising candidates were then taken through four additional tests, involving confirmation of effects on additional downstream target genes, characterization of phenotypes when expressed under additional Gal4 drivers, confirmation of phenotypes with additional, independent UAS-RNAi lines, and characterization of genetic interactions with known pathway components. Based on these experiments, a single gene, *Zyx102* (*Zyx*) [11],[12], which is located at 102F7 near the tip of the fourth chromosome, was identified as a novel component of the Fat-Hippo signaling pathway.

### *Zyx* Is Required for Hippo Signaling

**Figure 1:**
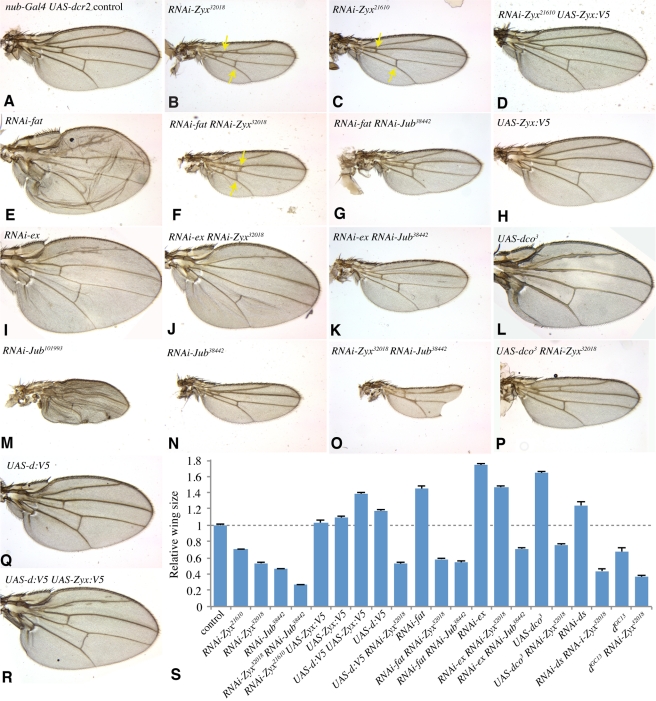
Zyx and Jub influence wing growth. All panels show wings from male adult flies with *nub-Gal4 UAS-dcr2*, and (A) no additional transgenes (control), (B) *UAS-RNAi-Zyx^32018^*, (C) *UAS-RNAi-Zyx^21610^*, (D) *UAS-RNAi-Zyx^2160^ UAS-Zyx:V5*, (E) *UAS-RNAi-fat*, (F) *UAS-RNAi-fat UAS-RNAi-Zyx^32018^*, (G) *UAS-RNAi-fat UAS-RNAi-Jub^38442^*, (H) *UAS-Zyx:V5*, (I) *UAS-RNAi-ex*, (J) *UAS-RNAi-ex UAS-RNAi-Zyx^32018^*, (K) *UAS-RNAi-ex UAS-RNAi-Jub^38442^*, (L) *UAS-dco^3^*, (M) *UAS-RNAi-Jub^101993^*, (N) *UAS-RNAi-Jub^38442^*, (O) *UAS-RNAi-Zyx^32018^ UAS-RNAi-Jub^38442^*, (P) *UAS-dco^3^UAS-RNAi-Zyx^32018^*, (Q) *UAS-d:V5*, and (R) *UAS-d:V5 UAS-Zyx:V5*. Yellow arrows point to cross-veins. (S) Average sizes for wings of the indicated genotypes, normalized to the average wing size in controls. 9–12 wings were measured per genotypes; error bars show s.e.m. Even modest differences in wing size were statistically significant (e.g., the 9% increase in UAS-Zyx:V5 versus control is significant by pairwise *t* test, *p*<0.0005).

Reduction of *Zyx* in the developing wing disc, under *nub-Gal4* control (Figure S1A), results in adult flies with small wings (Figure 1A–C,S). Similar phenotypes were observed using two different RNAi lines, although *NIG-32018R3* (*RNAi-Zyx^32018^*), the line identified in our original screen, has slightly stronger phenotypes. Hippo signaling also regulates leg growth, and depletion of *Zyx* in developing legs results in shorter legs with fewer tarsal segments (Figure S1I,J). In addition to observing similar phenotypes with two independent RNAi lines, confirmation that the phenotypes observed result specifically from reduction of *Zyx* was provided by the observation that over-expression of *Zyx* from a UAS transgene rescued the RNAi phenotypes (Figure 1D,S). We also confirmed by Western blotting that that *Zyx* RNAi reduced Zyx protein levels (Figure S1K).

**Figure 2:**
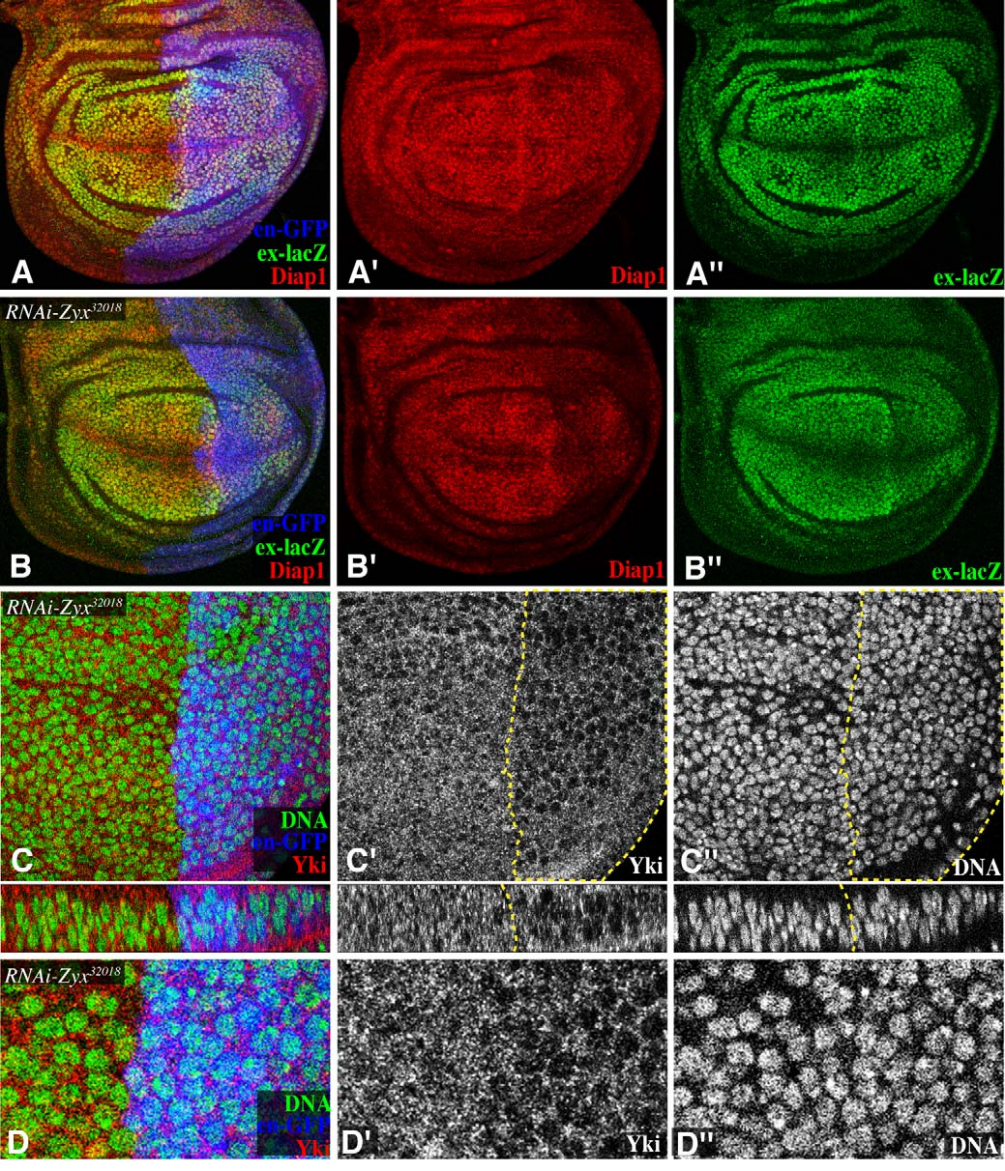
Zyx influences Yki activity in wing discs. (A–D) show third instar wing imaginal discs. In this and subsequent figures, panels marked by prime symbols show individual channels of the stain to the left. Discs in (A,B) are stained for Diap1 (red) and *ex-lacZ* (green), with posterior cells marked by GFP (blue), and have *en-Gal4 UAS-dcr2 UAS-GFP* transgenes, and (A) no additional transgenes (control), (B) *UAS-RNAi-Zyx^32018^*. (C,D) *en-Gal4 UAS-RNAi-Zyx^32018^ UAS-dcr2 UAS-GFP*, stained for Yki (red/white) and DNA (Hoechst, green/white) with posterior cells marked by GFP (blue) or demarcated by the dashed line. (C) Upper panels show a horizontal section; lower panels show a vertical section. (D) Higher magnification of a portion of the image shown in (C).

Many different genes and pathways affect organ growth. To investigate the potential connection between *Zyx* and the Hippo pathway, we examined the expression of downstream target genes in wing discs in which *Zyx* was depleted by RNAi. As downstream targets we employed reporters of *expanded* (*ex*) expression (*ex-lacZ*) and *th* expression (*th-lacZ*, Diap1). When *Zyx* was depleted from posterior cells using *en-Gal4*, *ex-lacZ*, *th-lacZ*, and Diap1were all reduced (Figures 2A,B, S2A). Hippo signaling regulates transcription by controlling the sub-cellular localization of Yki: activation of Hippo signaling promotes cytoplasmic localization of Yki, whereas inactivation of Hippo signaling allows nuclear localization of Yki, which corresponds to Yki activation [22],[23]. *Zyx* RNAi reduced nuclear Yki. This effect was subtle at late third instar, when levels of Yki in the nucleus are already low, but was evident in younger wing discs, which have higher levels of nuclear Yki (Figure 2C,D). The decreased expression of Hippo pathway target genes, together with the reduction in nuclear Yki, identifies *Zyx* as a regulator or component of the Hippo pathway. The Hippo pathway is generally thought of as a negative regulator of growth and gene expression, because most genes in the pathway act as tumor suppressors and negatively regulate the activity of Yki. *Zyx*, by contrast, is positively required for Yki activity and organ growth.

### *Zyx* Acts Genetically Within the Fat-Hippo Pathway

**Figure 3:**
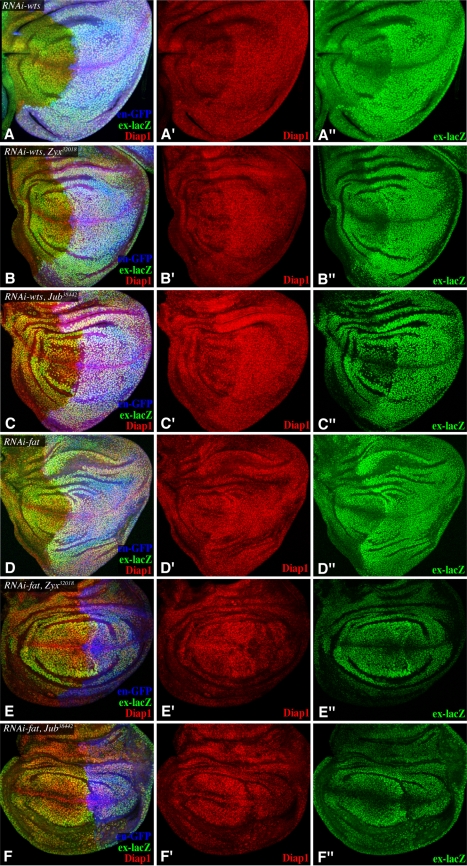
Epistatic relationship of *Zyx* and *Jub* to *wts* and *fat.* Wing imaginal discs, stained for Diap1 (red) and *ex-lacZ* (green), with posterior cells marked by GFP (blue), and with *en-Gal4 UAS-dcr2 UAS-GFP* transgenes, and (A) *UAS-RNAi-wts*, (B) *UAS-RNAi-wts UAS-RNAi-Zyx^32018^*, (C) *UAS-RNAi-wts UAS-RNAi-Jub^38442^*, (D) *UAS-RNAi-fat*, (E) *UAS-RNAi-fat UAS-RNAi-Zyx^32018^*, and (F) *UAS-RNAi-fat UAS-RNAi-Jub^38442^*.

To position the genetic requirement for *Zyx* within the Hippo pathway, we performed a series of epistasis tests. RNAi lines targeted against several different tumor suppressor genes within the pathway (*fat*, *ds*, *ex*, *wts*, *hpo*, and *mats*), each of which phenocopy their respective mutants, were examined in combination with *Zyx* RNAi lines. The immediate upstream regulator of Yki is *wts*. Expression of a *wts* RNAi line under *nub-Gal4* or *en-Gal4* control is lethal at late third instar, but imaginal discs can be recovered and analyzed before lethality. Consistent with the expected de-repression of Yki, expression of *wts* RNAi resulted in upregulation of *ex* and Diap1 expression (Figure 3A). This upregulation of *ex* and Diap1 was not suppressed by *Zyx* RNAi (Figure 3B); hence, *wts* is epistatic to *Zyx*. Wts activity is directly regulated by a kinase, Hippo (Hpo), and a co-factor, Mats, and *hpo* and *mats* were also epistatic to *Zyx* (Figure S3A–D). These observations imply that Zyx acts upstream of Wts.

**Figure 4:**
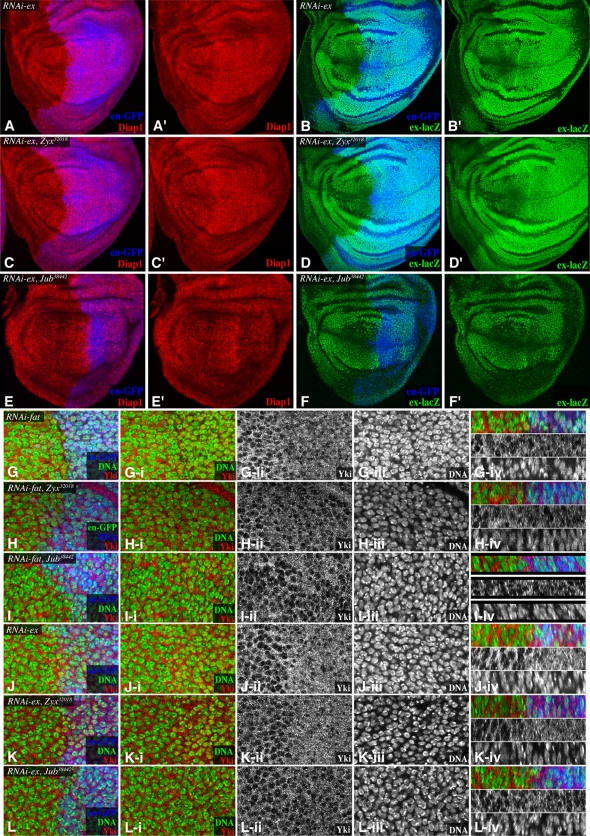
Epistatic relationship of *Zyx* and *Jub* to *ex*, and influence on Yki localization. Wing imaginal discs, stained for Diap1 (red) and *ex-lacZ* (green), with posterior cells marked by GFP (blue), and with *en-Gal4 UAS-dcr2 UAS-GFP* transgenes, and (A,B) *UAS-RNAi-ex*, (C,D) *UAS-RNAi-ex UAS-RNAi-Zyx^32018^*, (E,F) *UAS-RNAi-ex UAS-RNAi-Jub^38442^*. (G–L) show close-ups of portions of discs stained for Yki (red/white) and DNA (Hoechst, green/white) with posterior cells marked by GFP (blue), expressing *en-Gal4 UAS-dcr2 UAS-GFP* transgenes, and (G) *UAS-RNAi-fat*, (H) *UAS-RNAi-ex*, (I) *UAS-RNAi-fat UAS-RNAi-Zyx^32018^*, (J) *UAS-RNAi-ex UAS-RNAi-Zyx^32018^*, (K) *UAS-RNAi-fat UAS-RNAi-Jub^38442^*, and (L) *UAS-RNAi-ex UAS-RNAi-Jub^38442^*. Panels marked (i) show Yki and DNA, (ii) show Yki, (iii) show DNA, and (iv) show vertical sections, with triple stain at top, Yki in the middle, and DNA at bottom.

Upstream branches of Hippo signaling have been characterized in *Drosophila* as Fat-dependent, Ex-dependent, or Mer-dependent. In the developing wing, *fat* and *ex* make substantial contributions to Yki regulation, whereas *Mer* has a lesser role [6],[24]–[27]. Thus, we investigated the relationship between the requirement for *Zyx* and those for *fat* and *ex*. Expression of *fat* or *ex* RNAi throughout the wing, under *nub-Gal4* control, results in overgrown wings (Figure 1E,I,S). Strikingly, the wing overgrowth phenotype associated with depletion of *fat* was suppressed by *Zyx* RNAi, resulting in adult wings of similar size to those of animals that only expressed *Zyx* RNAi (Figure 1B,F,S). This epistasis of *Zyx* to *fat* was also visible at the level of target gene expression (Figure 3D,E) and the subcellular localization of Yki (Figure 4G,H). *Zyx* is also epistatic to the Fat ligand *ds* (Figures 1S, S1C,D). These observations imply that *Zyx* acts downstream of *fat*.

The *ex* RNAi phenotype, by contrast, was only slightly affected by *Zyx* RNAi, as the wings of *Zyx ex* double RNAi animals remained overgrown (Figure 1J,S). Moreover, *ex* was epistatic to *Zyx* for effects on downstream target gene expression and Yki localization (Figure 4A–D,J,K). Together, these observations indicate that *Zyx* specifically affects Fat-Hippo signaling and has little effect on Ex-Hippo signaling.

To refine our placement of *Zyx* within Fat-Hippo signaling, we examined requirements for *Zyx* relative to additional pathway components. *dco* encodes a kinase that phosphorylates the Fat cytoplasmic domain and participates in Fat-Hippo signaling [6],[28],[29]. The requirement for Dco within Fat signaling is uncovered by expression of an antimorphic isoform, Dco^3^. Expression of Dco^3^ induces wing overgrowth (Figure 1L) [29]. This overgrowth is suppressed by *Zyx* RNAi, suggesting that *Zyx* acts downstream of *dco* (Figure 1P,S).

Like *Zyx*, *dachs* is required for normal wing and leg growth and acts genetically downstream of *fat* and *dco* but upstream of *warts* [6]–[8]. To examine the genetic relationship between *Zyx* and *dachs*, we took advantage of the observation that over-expression of Dachs can promote wing overgrowth (Figure 1Q) [7]. This overgrowth was completely suppressed by *Zyx* RNAi (Figures 1S, S1G), as was the influence of Dachs over-expression on *ex-lacZ* expression (Figure S4A,B). Thus, Zyx is required for Dachs-promoted activation of Yki. Over-expression of Zyx resulted in a mild wing overgrowth on its own (9% increase in wing area, Figure 1H,S), and synergized with Dachs over-expression, resulting in enhanced wing overgrowth (Figure 1R,S). Together, these observations suggest that the functions of Zyx and Dachs in regulating growth are closely linked. However, the observation that *Zyx* depletion could enhance the small wing phenotype of a putative null allele of *dachs* (Figures 1S, S1E,F) [7] implies that Zyx also has some Dachs-independent influence on growth.

**Figure 5:**
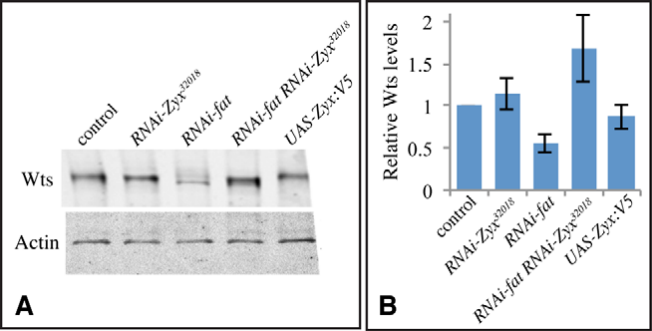
Wts Western blots. (A) Western blot on lysates of third instar wing discs from *tub-Gal4 UAS-dcr2* (control), *tub-Gal4 UAS-dcr2 UAS-RNAi-Zyx^32018^, tub-Gal4 UAS-dcr2 UAS-RNAi-fat, tub-Gal4 UAS-dcr2 UAS-RNAi-fat UAS-RNAi-Zyx^32018^*, and tub-Gal4 UAS-dcr2 UAS-Zyx:V5, probed with anti-Wts and anti-Actin antisera, as indicated. Similar amounts of total protein were loaded in each lane. (B) Quantitation of relative Wts protein levels in wing imaginal disc lysates. Wts and Actin band intensities were measured. To enable comparison across multiple blots, the Wts:Actin ratios were normalized to that detected in the control samples, which was set at 1. The histogram shows the average normalized ratios from five independent blots, error bars indicate s.e.m.

Fat exerts a post-transcriptional influence on the levels of Wts protein [6]. The genetic placement of *Zyx* upstream of *wts* and within the Fat branch of the pathway suggested that *Zyx* might also affect Wts levels. Indeed, *Zyx* RNAi completely suppressed the reduction in Wts levels associated with *fat* RNAi (Figures 5A,B, S2B). Thus, *Zyx* is genetically required for the mechanism that links Fat activity to the regulation of Wts protein levels. The influence of *fat* on Warts levels also requires *dachs* [6]. *Zyx* RNAi did not detectably affect Dachs localization (Figure S4D,E), nor did *Zyx* RNAi affect Fat localization (Figure S5E,F). In addition to its effects on Wts, *fat* mutation also decreases the levels of Ex at the sub-apical membrane [30]–[33]. *Zyx* RNAi was not able to reverse this effect of *fat* on Ex levels (Figure S5G–N). Depletion of *Zyx* in the wing disc also did not have visible effects on F-actin (Figure S5O,P).

In addition to regulating transcription, Fat also regulates planar cell polarity (PCP) (reviewed in [1],[5]). PCP in the adult wing is manifest in the orientation of wing hairs, which point distally. The anterior, proximal wing is particularly sensitive to Fat-PCP signaling, and *fat* RNAi results in strong PCP phenotypes in this region, including reversals of hair polarity (Figure S1M). PCP phenotypes have also been described in this region of *dachs* mutant wings [34]. *Zyx* RNAi, by contrast, had no detectable effect on wing PCP (Figure S1N), and a PCP phenotype was also still detected in *fat Zyx* double RNAi wings (Figure S1O). Genes previously identified as influencing Fat-PCP signaling (i.e., *fat*, *ds*, *fj*, *app*, *dachs*, *lft*) also influence cross-vein spacing. *Zyx* RNAi wings sometimes have extra cross-veins, but by contrast to *dachs* mutants, the anterior and posterior cross-veins remain well-separated in *Zyx* RNAi flies (Figure 1B,C), and the influence of *fat* on cross-vein spacing is not suppressed by *Zyx* (Figure 1F). Our observations suggest that *Zyx* is specifically required for Fat-Hippo signaling, and not for Fat-PCP signaling, although because *Zyx* RNAi might not completely eliminate Zyx, we cannot exclude the possibility that low levels of Zyx are sufficient for PCP, but not for Hippo signaling.

### Localization of Zyx to the Sub-Apical Membrane

**Figure 6:**
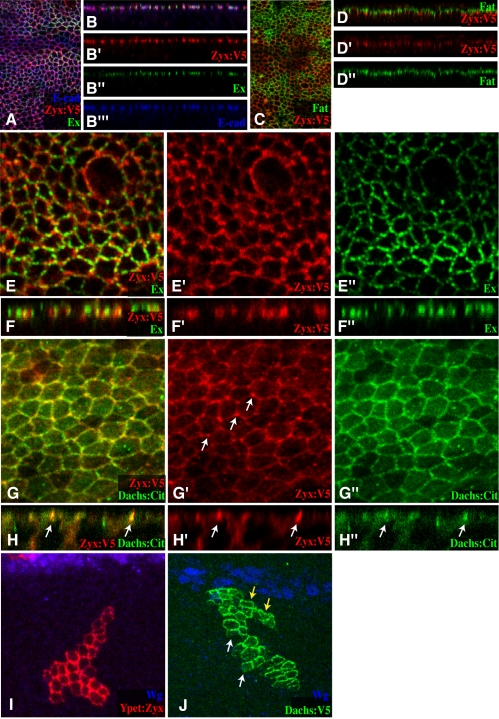
Zyx localization in wing imaginal discs. All panels show Zyx localization in wing discs, based on UAS-Zyx:V5 (anti-V5, red) or UAS-Ypet:Zyx (red) transgenes. (A,B) Zyx localization versus E-cad (blue) and Ex (green) in an apical horizontal section (A) and vertical sections (B). (C,D) Zyx localization versus Fat (green) in an apical horizontal section (C) and vertical sections (D). (E,F) Close-up of Zyx localization versus Ex (green) in an apical horizontal section (E) and vertical sections (F). (G,H) Close-up of Zyx localization versus Dachs (using Dachs:Citrine, green) in an apical horizontal section (G) and vertical sections (H). (I) Close-up of Zyx localization in a clone. Zyx staining does not exhibit a proximal-distal bias. The stronger staining in the center of the clone presumably reflects the fact that this staining comes from two adjacent cells. (J) Close-up of Dachs localization in a clone. Dachs staining is strong on the distal side (yellow arrows) and weak on the proximal side (white arrows). Proximal-distal orientation is evidenced in these panels by Wg expression (blue) along the dorsal-ventral compartment boundary.

As our anti-Zyx sera did not work for immunostaining, we made use of a V5-tagged UAS transgene that rescues the *Zyx* RNAi phenotype (Figure 1) to investigate the subcellular localization of Zyx in imaginal discs. We also examined a UAS-Ypet:Zyx transgene [35]. Although our localization studies are subject to the caveat that Zyx protein was over-expressed, the two different tagged Zyx proteins have similar localization profiles, and similar localization profiles were observed using different Gal4 drivers. Zyx was preferentially localized to the sub-apical membrane of disc cells (Figure 6). This sub-apical membrane staining was at the same apical-basal position as E-cadherin (E-cad), and just basal to Fat (Figure 6A–D). This is similar to the membrane localization of Dachs [7]. Indeed, when we compared Zyx and Dachs localization, using epitope-tagged constructs, we observed that the membrane staining is at the same apical-basal position and that they partially co-localize (Figure 6G,H). A distinguishing feature of Dachs localization is its polarization within the plane of the epithelium, which occurs in response to the Fj and Ds gradients (Figure 6J) [7],[9]. Zyx, by contrast, is not planar-polarized (Figure 6I); hence, Zyx and Dachs are expected to overlap on only one side of wing disc cells. A distinguishing feature of Zyx staining is that it often displays puncta of larger, more intense staining at the vertices where three cells meet (Figure 6G). Intriguingly, Ex protein also displays uneven staining, but Ex puncta are partially complementary to Zyx puncta (Figure 6E,F). These observations suggest that even though Ex and Zyx localize to a similar apical-basal position, they assemble into distinct protein complexes. Dachs localization was not visibly affected by RNAi of *Zyx* (Figure S4E), nor was Zyx localization affected by mutation of *dachs* (Figure S5B), which indicates that neither protein depends upon the other for its localization. Zyx localization was also not visibly affected by mutation or RNAi of *fat, ex*, or *wts* (Figure S5 and unpublished data).

### Dachs Promotes Zyx-Wts Binding

**Figure 7:**
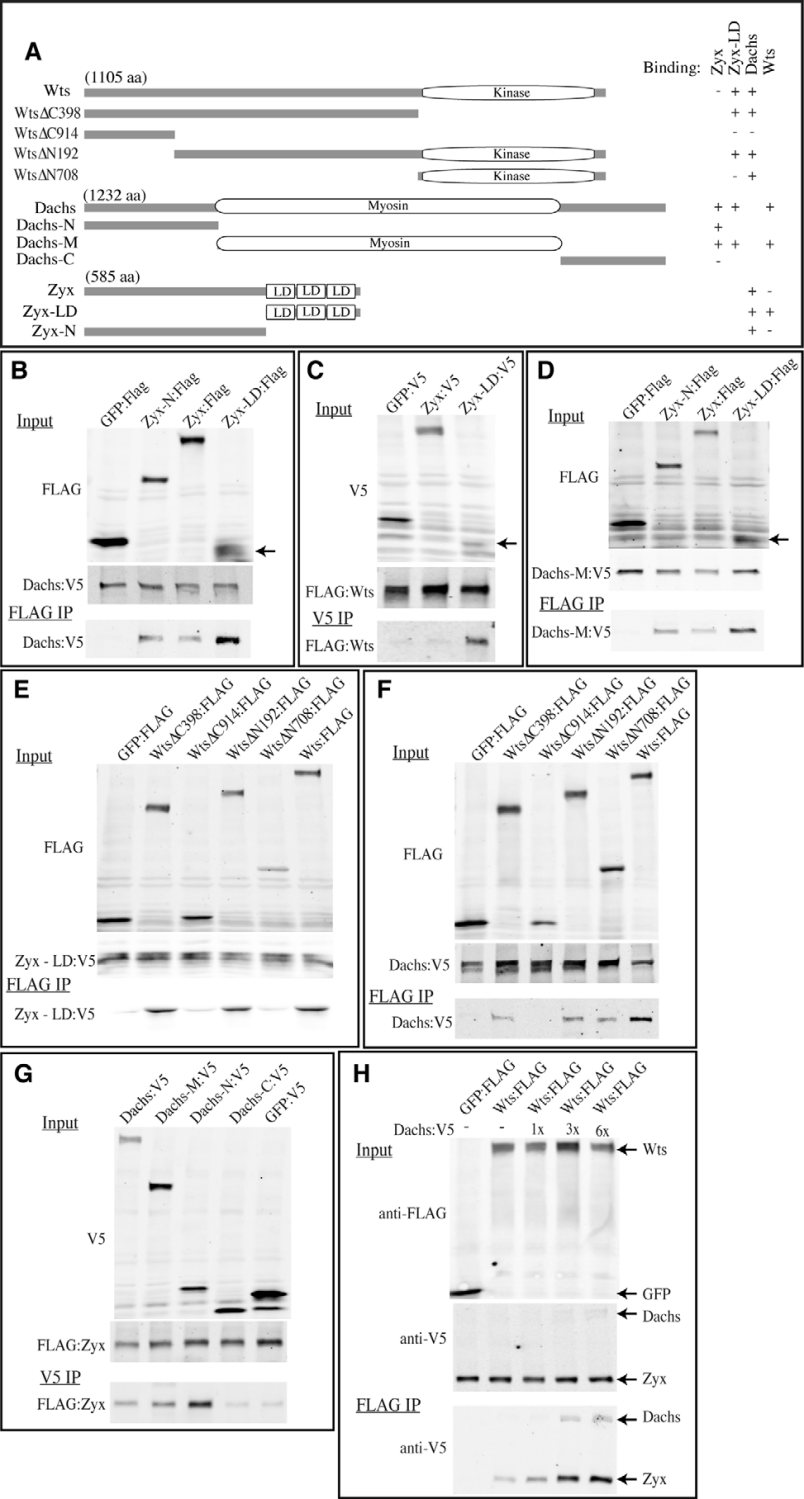
Binding amongst Zyx, Dachs, and Wts. (A) Schematic of Wts, Dachs, and Zyx proteins, and the constructs used to map interaction domains. LD indicates Lim domain. Binding interactions are summarized to the right; + indicates strong binding, and − indicates weak or no binding. (B–G) show Western blots on co-immunoprecipitation experiments, with upper two blots indicating the relative amount of protein in the lysates used for the experiments and the lower panel indicating the material co-precipitated by the indicated antibody. GFP serves as a negative control. In (B–D) arrow identifies the Zyx-LD:FLAG polypeptide, and other bands in this lane are non-specific background detected by the antibodies. (B) Co-precipitation of V5-tagged Dachs with the FLAG-tagged proteins indicated at top. (C) Co-precipitation of FLAG-tagged Wts with the V5-tagged proteins indicated at top. (D) Co-precipitation of V5-tagged Dachs myosin domain with the FLAG-tagged proteins indicated at top. (E) Co-precipitation of V5-tagged Zyx-LD polypeptide with the FLAG-tagged proteins indicated at top. (F) Co-precipitation of V5-tagged Dachs with the FLAG-tagged proteins indicated at top. (G) Co-precipitation of FLAG-tagged Zyx with the V5-tagged proteins indicated at top. (H) Co-precipitation of V5-tagged Dachs and Zyx with the FLAG-tagged proteins indicated at top, in the presence of increasing amounts of Dachs:V5, as indicated. 1x indicates that equal amounts of pUAS-Zyx:V5 and pUAS-dachs:V5 plasmids were used, and 3x and 6x indicate corresponding increases in amounts of pUAS-dachs:V5 plasmid transfected. Note that in the absence of Dachs, no binding between full-length Zyx and Wts was detected when proteins were precipitated using anti-V5 beads and GFP:V5 was used as a negative control (panel C), but weak binding was detected when proteins were precipitated using anti-FLAG beads and GFP:FLAG was used as a negative control (H).

The similar genetic requirements for *Zyx* and *dachs* in Fat-Hippo signaling, together with their partial co-localization in imaginal discs, raised the possibility that Zyx and Dachs might interact. This was investigated by expressing tagged isoforms in cultured *Drosophila* S2 cells and assaying for physical interactions through co-immunoprecipitation. Indeed, Zyx and Dachs could be specifically co-precipitated from S2 cells (Figure 7B). This observation suggests that Dachs and Zyx can interact directly, although it is also possible that they interact indirectly through a larger complex including endogenously expressed proteins within S2 cells.

As Dachs can also associate with Warts in co-immunoprecipitation assays [6], and both *Zyx* and *dachs* are required for the *fat*-dependent regulation of Wts levels, we also investigated binding between Zyx and Wts. When tagged full-length proteins were co-expressed in S2 cells, little or no Zyx-Wts co-precipitation was detected (Figure 7C,H). However, in addition to their role in Hippo signaling, functions for LATS proteins have also been identified in mitosis, and LATS1 has been localized to the mitotic apparatus [36],[37]. In the context of a study of mitotic functions of LATS1, it was reported that the C-terminus of human Zyxin, including the LIM domains, could bind to human LATS1, even though full-length Zyxin did not bind [36]. When we expressed a C-terminal polypeptide comprising the LIM domains of Zyx (Zyx-LD) in S2 cells, only very low levels of protein could be detected (Figure 7B–D). Nonetheless, this C-terminal polypeptide bound efficiently to Wts (Figure 7C). Thus, the LIM domains of Zyx can associate with Wts, but this association is normally inhibited within full-length Zyx.

The discovery of this latent ability of Zyx to bind Wts, together with our discovery of Zyx-Dachs binding, and previous identification of Dachs-Wts binding [6], indicates that Dachs, Zyx, and Wts each have the ability to bind to one another. To gain further insight into complex formation among these proteins, we mapped their interaction domains. Wts bound to the LIM domains of Zyx. Dachs, by contrast, bound most strongly to the C-terminal LIM domains but also bound to the N-terminal half of Zyx (Figure 7B). Dachs contains a large central myosin motor domain and could bind to both Zyx and Wts through this motor domain (Figure 7D,G and unpublished data). Zyx-LD bound to Wts through a region N-terminal to the Wts kinase domain (Figure 7E). Dachs bound both to this region and also to the Wts kinase domain (Figure 7F). Thus, Zyx, Dachs, and Wts interact with each other through partially overlapping domains.

**Figure 8:**
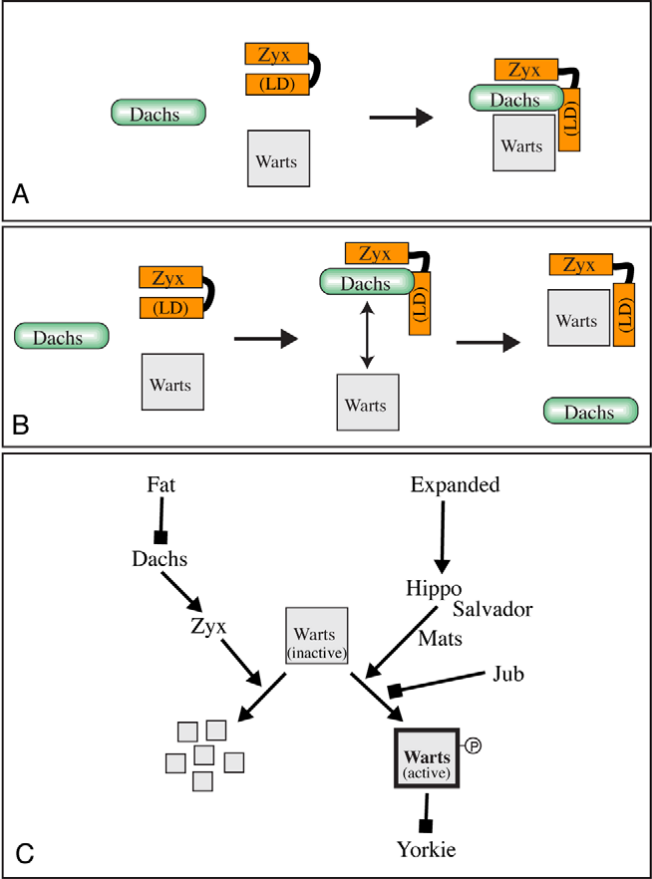
Models for Zyx function in Fat-Hippo signaling. (A) Dachs might bridge Zyx and Wts within a trimeric complex; the simplest version of this model (stoichiometric amounts) would predict that in order for Zyx to be co-precipitated with Wts, Dachs and Zyx levels within the complex would have to be equivalent, which was not observed. (B) Dachs might induce a conformational change in Zyx (either directly through binding as shown or by recruiting other factors), exposing the LIM domains and enabling them to bind Wts. (C) illustrates the distinct roles of the LIM-domain proteins Zyx and Jub in Hippo signaling. Zyx influences the levels of Wts protein, presumably by promoting Wts degradation, whereas Jub inhibits Wts activation. The ability of Zyx to interact with Wts is regulated by Dachs, and Dachs in turn is regulated by Fat.

To assay for potential sequential, cooperative, or competitive interactions amongst Zyx, Dachs, and Wts, we examined binding interactions when all three proteins were co-expressed together in S2 cells. A key feature of Zyx's interactions with Wts is that full-length Zyx does not bind efficiently to Wts, but the LIM domains do. However, we found that Dachs enhanced the co-precipitation of full-length Zyx with Wts (Figure 7H). Two basic models for this stimulation of Zyx-Wts association by Dachs can be envisioned: (a) Dachs might bridge Wts and Zyx within a Wts-Dachs-Zyx complex, or (b) Dachs might trigger a conformational change in Zyx that reveals the latent Wts-binding activity of the Zyx LIM domains (Figure 8A,B). By employing V5 epitope tags on both Zyx and Dachs, and assaying their co-precipitation with FLAG-tagged Wts, we could directly compare their association with Wts. A simple trimeric complex model (e.g., one subunit each of Zyx, Wts, and Dachs) would predict that Zyx and Dachs should be present within the Wts trimeric complex at equal levels. However, we found instead that Zyx could be much more abundant in Wts complexes than Dachs (Figure 7H). This suggests that rather than remaining stably associated with Zyx and Wts in a trimeric complex, Dachs is able to stimulate a conformational change in Zyx that exposes the LIM domains and enables them to bind Wts. Consistent with this model, Dachs stimulated Zyx binding to Wts but did not stimulate the binding of Zyx-LD to Wts (Figure S6A).

### The Requirement for *Jub* in Hippo Signaling Is Distinct from that of *Zyx*

Zyx is a *Drosophila* member of a group of cytoskeletal-associated proteins with three C-terminal LIM domains [38]. These comprise two families: the Zyxin family, which in vertebrates includes Zyxin, Lipoma preferred partner (LPP), and Thyroid-receptor interacting protein 6 (TRIP6), and the Ajuba family, which in vertebrates includes Ajuba, LIM domain containing 1 (LIMD1), and Wilms tumor protein 1-interacting protein (WTIP). *Drosophila* have a single member of each family; Zyx is a member of the Zyxin family, and Ajuba LIM protein (Jub) is a member of the Ajuba family. Ajuba has been reported to interact with a human homologue of Warts, LATS2 [39], and Das Thakur et al. (2010) recently reported that mutation or RNAi-mediated depletion of *Jub* reduces growth through interactions with the Hippo pathway, and through genetic and protein interaction experiments positioned Jub as a regulator of Wts [40]. In agreement with this, we found that RNAi-mediated depletion of *Jub* reduces wing growth (Figure 1M,N,S), expression of Hippo pathway target genes, and nuclear Yki (Figure S7), and that *wts* is epistatic to *Jub* (Figure 3C). As for *Zyx*, depletion of *Jub* did not detectably influence wing hair PCP (Figure S1P,K).

The determination that *Zyx* and *Jub* are each genetically required for Hippo signaling suggests that they have distinct functional roles, and consistent with this, we observed that over-expression of Zyx could not rescue *Jub* RNAi phenotype (Figure S1H) and that *Zyx Jub* double RNAi induced an even greater reduction of wing size than when they were expressed individually (Figure 1O,S). Das Thakur et al. (2010) did not address the relationship of Jub to upstream regulators of Hippo signaling. Intriguingly, we found that depletion of *Jub* suppressed both *fat* and *ex* phenotypes. This suppression was evident upon examination of adult wings (Figure 1G,K,S), expression of downstream target genes in wing discs (Figures 3F, 4E,F), and the sub-cellular localization of Yki (Figure 4I,L). Thus, by contrast to *Zyx*, which functions specifically within Fat-Hippo signaling, *Jub* is required for both Ex-Hippo and Fat-Hippo signaling. This observation confirms that these two LIM-domain proteins have functionally distinct roles within the Hippo pathway.

The distinct genetic role of *Jub* in Hippo signaling is also reflected in distinct binding interactions. By contrast to the crucial role of Dachs in stimulating binding between full-length Zyx and Wts, full-length Jub binds efficiently to Wts, and full-length vertebrate homologues of Jub bind to LATS proteins [39],[40]. Moreover, Jub bound only very weakly Dachs (Figure S6B). Thus, although Zyx and Jub share the ability to associate with Wts through their LIM domains, both genetic and biochemical studies indicate that the regulation and consequences of these LIM-domain-Wts interactions are distinct.

## Discussion

Our characterization of Zyx identifies a role for it as a novel and integral component of the Hippo pathway, which is required for the Fat branch, but not the Ex branch, of Hippo signaling. Unlike most previously identified components, loss of Zyx reduces the activity of the key transcriptional effector of the pathway, Yki, and consequently its loss reduces organ growth. Genetic epistasis experiments position the requirement for *Zyx* in between *fat* and *wts*, and concordant protein binding experiments identify a Dachs-stimulated ability of Zyx to bind Wts protein. We infer that this association of Zyx with Wts then downregulates Wts, at least in part, by targeting it for degradation.

Zyx localizes to the sub-apical membrane independently of Fat or Dachs. Since Fat regulates the localization of Dachs [7], this regulated localization provides a mechanism by which Fat could modulate the interaction of Dachs with Zyx (although we note that Fat might affect the activity of Dachs in addition to affecting its localization). Since Dachs stimulates Zyx-Wts binding, this regulated localization provides a means for Fat signaling to modulate Zyx-Wts binding. We infer that Dachs effects a conformational change in Zyx, as in the absence of Dachs a Zyx LIM-domains polypeptide binds efficiently to Wts, whereas full-length Zyx binds poorly. Intriguingly, the association of vertebrate homologues of Zyx and Warts can also be post-translationally regulated, as the ability of the LIM domains of human LATS1 to bind Zyxin is masked within full-length Zyxin, but uncovered by Cdc2-mediated phosphorylation, presumably due to conformational change [36]. We hypothesize that the ability of Dachs to bind to both the N-terminus and the LIM domains of Zyx enables it to effect a conformational change in Zyx, resulting in an open configuration that can bind to Wts (Figure 8B). It is also possible that Dachs binding stimulates a post-translational modification of Zyx to induce a conformational change.

Prior studies identified two mechanisms by which Fat signaling could influence Yki activity, as *fat* mutation reduces both the levels of Wts protein [6] and the amount of Ex at the sub-apical membrane [31]–[33]. It has not been possible to completely uncouple these two pathways for Fat-Hippo signaling, although the observation that over-expression of Wts can efficiently suppress *fat* overgrowth phenotypes, but only partially suppresses *ex* overgrowth phenotypes [30], suggested that the influence of Fat on Wts levels might be more critical. Analysis of the influence of Zyx on Ex is complicated by its influence on *ex* transcription, but our observation that reduction of *Zyx* does not appear to suppress the influence of *fat* on Ex staining, even though it does suppress the influence of *fat* on Wts levels, also suggests that the influence of Fat on Wts levels might be more critical than its effects on Ex. Intriguingly, mutation of *dachs* did suppress the influence of *fat* on Ex levels [30]. Although it is possible that this difference between *dachs* and *Zyx* results from technical differences in the experimental paradigms (e.g., mutant clones versus RNAi), it is also possible that *dachs* can influence Ex levels independently from its association with Zyx.

The discovery of the Fat-specific effect on Wts levels, by contrast to the Hippo-pathway-mediated effect on Wts kinase activity, established the concept of distinct mechanisms for regulating Wts—one that affects Wts levels and another that affects Wts activity [6]. Our identification of distinct genetic requirements for *Zyx* and *Jub* provide further support for this concept. As *Jub* is equally required for both Fat-Hippo and Ex-Hippo signaling and acts genetically between *hippo* and *wts* [40], Jub appears to inhibit Wts activation. In our working model (Figure 8C), the epistasis of *Jub* to *fat* could be explained by an increased activity of residual Wts, which then acts catalytically to repress Yki activity. *Zyx* is required for the influence of *fat* on Wts levels. We note that when measured within a whole tissue lysate, Wts levels are only reduced to approximately half their normal levels. However, as Wts appears to function within multi-protein complexes, including some components that can localize preferentially to the sub-apical membrane [41],[42], we hypothesize that Fat signaling affects a discrete pool of Wts within a complex at the membrane that is crucial for Hippo signaling, whereas there might be additional pools of Wts within the cell that are unaffected. We also note that while we clearly see effects on Wts protein levels, our results do not exclude the possibility that Fat signaling also influences Wts activity.

Our characterization of Zyx and Jub also provides new tools for analyzing critical steps in Hippo signaling. For example, in addition to influencing Hpo and Wts kinase activity, it has been observed that Ex can bind directly to Yki and that when Ex is over-expressed it can repress Yki through a mechanism that involves direct sequestration of Yki, rather than regulation of Yki phosphorylation [43],[44]. Because this direct repression mechanism was based on over-expression experiments, the extent to which it contributes to normal Yki regulation in vivo remained uncertain. The observations that *Jub* acts genetically upstream of *wts*, yet is required for *ex* phenotypes, suggests that Ex regulates Yki principally through its effects on Wts activity, rather than through direct interaction with Yki.

The ability of Zyx LIM domains to interact with Wts is conserved in their human homologues [36]. Although the functional significance of this interaction in vertebrates has not yet been established, our observations raise the possibility that the oncogenic effects of human *LPP* mutations [13] could be due to an ability of these aberrant LPP fusion proteins to negatively regulate LATS proteins, resulting in inappropriate activation of YAP or TAZ.

One of the most intriguing aspects of Zyxin family proteins is their role in mediating effects of mechanical force on cell behavior [18]. Zyxin family proteins can localize to focal adhesions of cultured fibroblasts, and this localization is modulated by mechanical tension [15],[18],[45]. The observation that increasing tension on stress fibers stimulates Zyxin accumulation at focal adhesions is intriguing in light of our observation that Zyx tends to accumulate at higher levels at intercellular vertices in imaginal discs, as these could be points of increased tension. As the association of unconventional myosins with F-actin can also be influenced by external force [46], our discovery of binding between a myosin protein (Dachs) and Zyx raises the possibility that other myosins might also interact with Zyxin family proteins, which could potentially influence either their tension-based recruitment or their activity.

Finally, we note that theoretical models of growth control in developing tissues have proposed that growth should be controlled by mechanical tension [47],[48], and direct evidence for mechanical effects on growth has been obtained in cultured cell models [49]. However, a mechanism for how this might be achieved has been lacking. Our discovery that Zyx, a member of a family of proteins implicated in responding to and transducing the effects of mechanical tension, is also a component of the Hippo signaling pathway, a crucial regulator of growth from *Drosophila* to humans, raises the intriguing possibility that Zyxin family proteins might form part of a molecular link between mechanical tension and the control of growth.

## Materials and Methods

### *Drosophila* Genetics

RNAi screening was conducted using lines from the NIG-Fly Stock Center (http://www.shigen.nig.ac.jp/fly/nigfly/index.jsp), which were crossed to *vg-Gal4 UAS-dcr2* or *pnr-Gal4 UAS-dcr2*. Those with growth phenotypes were then re-screened for effects on Diap1 and Wg expression in imaginal discs by crossing to *ci-Gal4 UAS-dcr2* or *en-Gal4 UAS-dcr2*. All crosses were carried out at 28.5 C to obtain stronger phenotypes. Approximately 1,200 lines were examined in the initial screen (Table S1).

Additional RNAi lines employed include *ds* [vdrc 36219], *fat* [vdrc 9396], *d* [vdrc 12555], *ex* [vdrc 22994], *Zyx* [NIG-32018R3], *Zyx* [vdrc 21610], *wts* [vdrc 9928], *wts* [NIG-12072R1], *mats* [vdrc 108080], *hpo* [vdrc 104169], *Jub* [vdrc 101993], and *Jub* [vdrc 38442]. The effectiveness of *fat* and *ex* RNAi is illustrated in Figure S3E,F. Both *Zyx* RNAi lines gave similar effects on growth and gene expression in combination with multiple Gal4 lines and also behaved similarly in epistasis tests. UAS lines employed include *UAS-dco^3^* [29],[48], *UAS-d:V5[9F]* and *UAS-d:V5* [50] [7], *UAS-d:citrine* [*28*] (B.K. Staley, unpublished), *UAS-Zyx:V5*, and *UAS-Ypet:Zyx* [35]. Gal4 lines employed include *Dll-Gal4*, *ex-lacZ en-Gal4 UAS-GFP/CyO;UAS-dcr2/TM6b*, *en-Gal4/CyO*; *th-lacZ UAS-dcr2/TM6b*, *ci-Gal4 UAS-dcr2* [*3*] */TM6b*, *w UAS-dcr2[X]; nub-Gal4[ac-62]*, *w; AyGal4 UAS-GFP/C yO;UAS-dcr2/TM6b*, *y w hs-FLP[122]; AyGal4 UAS-GFP/CyO*, *tub-Gal80^ts^/CyO,Act-GFP; tub-Gal4 UAS-dcr2/ TM6b*, *w; tub-Gal4/CyO-GFP.* MARCM clones were made by crossing *y w hs-FLP[122] tub-Gal4 UAS-GFP/FM7 ; tub-Gal80 FRT40A/CyO* to *fat^8^ FRT40A/CyO*, *ex^el^ FRT40A/CyO*, *d^GC13^ FRT40A/CyO* or *y^+^ FRT40A* (as a control) and *UAS-zyxin:V5*. Flp-out clones were made by crossing *y w hs-FLP[122]; AyGal4 UAS-GFP* to *UAS-zyxin:V5* or crossing *AyGal4; UAS-d:citrine* to *y w hs-FLP[122]; UAS-zyxin:V5*. Genetic interaction of *Zyx* and *dachs* was examined by recombining *nub-Gal4* with *d^GC13^* and crossing to *d^GC13^; RNAi-Zyx32018*.

Adult wing phenotypes were scored by crossing *UAS-dcr2; nub-Gal4* females to males of RNAi lines or Oregon-R males as a control. Wings of male progeny were photographed, all at the same magnification. For quantitation, between 9 and 12 wings per genotype were traced using NIH Image J, and wing areas were normalized to the average area in control males. Standard error of the mean (s.e.m.) and *t* tests were calculated using Graphpad Prism software.

### Histology

For analysis of gene expression in imaginal discs, *ex-LacZ en-Gal4 UAS-GFP; UAS-dcr2* females were crossed to RNAi line males, and larvae were kept at 28.5 C until dissection. For analysis of Zyx:V5 or Ypet:Zyx localization, expression was driven by *en-Gal4*, *AyGal4*, or *tub-Gal4*. Discs were fixed in 4% paraformaldehyde and stained using as primary antibodies: goat anti-ß-galactosidase (1∶1,000, Biogenesis), mouse anti-Diap1 (1∶200, B. Hay), rat anti-E-cad (1∶200, DSHB), guinea pig anti-Ex (1∶2000, R. Fehon), rat anti-Fat (1∶400) [29], mouse anti-V5 (1∶400, Invitrogen), mouse anti-Wg (1∶400, DSHB), and rabbit anti-Yki (1∶400) [22]. F-actin was stained using Alexa Fluor 546 phalloidin (1∶100, Invitrogen), and DNA was stained using Hoechst (Invitrogen).

### Plasmid Constructs

Details of plasmid construction are in Text S1.

### Co-immunoprecipitation and Western Blotting

Co-immunoprecipitation assays were performed as described previously [6]. Cell lysates were cleared using protein G beads (Sigma). Anti-V5 or anti-FLAG M2 beads (Sigma) were incubated with cell lysates overnight at 4°C, then washed six times with RIPA buffer and boiled in SDS-PAGE loading buffer. Primary antibodies used for blotting include rabbit anti-V5 (1;10,000, Bethyl), mouse anti-V5 (1∶10,000, Invitrogen), and mouse anti-FLAG M2 (1∶10,000, Sigma), and were detected using anti-mouse IRdye680 and goat anti-rabbit IRdye800 (1∶10,000, LiCor) and scanning on a LiCor Odyssey.

For analysis of Wts protein levels, *tub–Gal4 UASdcr2/ TM6b* females were crossed to *white (control)*, *RNAi-fat*, *RNAi-Zyx*, *RNAi-fat*; *RNAi-Zyx*, or *UAS-Zyx:V5* males, and wing discs were dissected from third instar larval progeny and lysed in RIPA buffer. Amounts loaded were adjusted to try to load equivalent amounts of total protein in each lane. Wts was detected using a published Wts anti-sera [6] at 1∶4,000. Protein bands were detected using anti-mouse IRdye680 and goat anti-rabbit IRdye800 (1∶10,000, LiCor) and scanning on a LiCor Odyssey. Bands were quantified using LiCor Odyssey software. Relative Wts levels were determined by comparison to bands detected by anti-Actin antibodies (mouse anti-Actin at 1∶5,000, Calbiochem). To enable the relative levels of Wts to be averaged across different blots, we normalized the ratios on each blot to that detected for the control lane, which was set as 1.

For confirmation of the influence of *Zyx* RNAi on Zyx protein levels, *tub–Gal4 UASdcr2/TM6b* females were crossed to *white (control)*, or *RNAi-Zyx^32018^*, and cultured at 29 C, and wing discs were dissected from third instar larval progeny and lysed in RIPA buffer. A rabbit anti-Zyx sera was used at a 1∶2,000 dilution, and subsequently the blot was re-probed with rabbit anti-actin (1∶10,000, Sigma). Fluorescent detection was performed as described above. Anti-Zyx sera was obtained by immunization of rabbits with a KLH conjugated peptide (KRRLDIPPKPPIKY), performed by Open Biosystems.

## Supporting Information

Figure S1 Additional characterization of the influence of Zyx and Jub on wing and leg growth and PCP. (A) Wing imaginal disc from *nub-Gal4 UAS-dcr2 UAS-GFP* larva; the *nub* expression domain is indicated by GFP expression (green); for reference Wg expression (red) is also shown. Panels (B–F) show wings from male adults flies with *nub-Gal4 UAS-dcr2*, and (B) no additional transgenes (control), (C) *UAS-RNAi-ds*, (D) *UAS-RNAi-ds UAS-RNAi-Zyx^32018^*, (E) *UAS-dachs:V5 UAS-RNAi-Zyx^32018^*, and (F) *UAS-Zyx:V5 UAS-RNAi-Jub^38442^*. Panels (G,H) show wings from male adults flies of (G) *d^GC13^ nub-Gal4* and (H) *d^GC13^ nub-Gal4 UAS-RNAi-Zyx^32018^.* (I) Leg from *Dll-Gal4 UAS-dcr2* adult male control. (J) Leg from *Dll-Gal4 UAS-dcr2 UAS-RNAi-Zyx^32018^* adult male. (K) Western blot on lysates of third instar wing discs from *tub-Gal4 UAS-dcr2* (control) and *tub-Gal4 UAS-dcr2 UAS-RNAi-Zyx^32018^* (*RNAi-Zyx^32018^*) probed with anti-Zyx and anti-Actin antisera, as indicated. Similar amounts of total protein were loaded in each lane. (L–Q) show close-ups of the anterior wing from male adults flies with *nub-Gal4 UAS-dcr2*, and (L) no additional transgenes (control), (M) *UAS-RNAi-fat*, (N) *UAS-RNAi-Zyx^32018^*, (O) *UAS-RNAi-fat UAS-RNAi-Zyx^32018^*, (P) *UAS-RNAi-Jub^38442^*, and (Q) *UAS-RNAi-Zyx^32018^ UAS-RNAi-Jub^38442^*. Blue arrows indicate normal polarity; red arrows indicate disturbed polarity.
(8.60 MB TIF)

Figure S2 Additional characterization of the influence of *Zyx* on Yki activity. (A) Third instar *en-Gal4 UAS-dcr2 UAS-RNAi-Zyx^32018^* wing imaginal disc, stained for *th-lacZ* (red), with posterior cells marked by Dcr2 (blue). (B) Western blot on lysates of third instar wing discs from *tub-Gal4 UAS-dcr2* control (+), *tub-Gal4 UAS-dcr2 UAS-RNAi-Zyx^32018^*, *tub-Gal4 UAS-dcr2 UAS-RNAi-fat*, *tub-Gal4 UAS-dcr2 UAS-RNAi-fat UAS-RNAi-Zyx^32018^*, and *UAS-Zyx:V5*, probed with anti-Wts. This panel shows the entire blot for the bands depicted in Figure 5A. The Wts band was identified based on its mobility and the observation that this band is decreased by *wts* RNAi. Numbers indicate the calculated mobilities of the size markers.
(1.46 MB TIF)

Figure S3 Additional characterization of the epistatic relationship of *Zyx* to the Hippo pathway. Wing imaginal discs, stained for *ex-lacZ* (green), with posterior cells marked by GFP (blue), and with *en-Gal4 UAS-dcr2 UAS-GFP* transgenes, and (A) *UAS-RNAi-mats*, (B) *UAS-RNAi-hpo*, (C) *UAS-RNAi-mats UAS-RNAi-Zyx^32018^*, (D) *UAS-RNAi-hpo UAS-RNAi-Zyx^32018^*, (E) *UAS-RNAi-fat*, and (F) *UAS-RNAi-ex*. Discs in (E and F) are also stained for anti-Fat (red, E) and anti-Ex (red, F). Both RNAi lines are highly effective, but the anti-Ex sera gives higher background staining.
(7.74 MB TIF)

Figure S4 Additional studies of *Zyx* epistasis and Zyx localization in wing imaginal discs. (A,B) Wing imaginal discs, stained for Wg (red) and *ex-lacZ* (green), with posterior cells marked by GFP (blue), and with *en-Gal4 UAS-dcr2 UAS-GFP* transgenes, and (A) *UAS-dachs:V5* or (B) *UAS-dachs:V5 UAS-RNAi-Zyx^32018^* transgenes. (C) *en-Gal4 UAS-dcr2 UAS-GFP UAS-Zyx:V5* wing imaginal disc, stained for *ex-lacZ* (green), with posterior cells marked by GFP (blue). (D,E) Close-ups of wing imaginal discs, stained for E-cad (red), showing clones of cells expressing Dachs:V5 (green), under *AyGal4* control, with *AyGal4 UAS-dcr2 UAS-dachs:V5* transgenes, and (D) no additional transgenes (control) or (E) *UAS-RNAi-Zyx^32018^*. Yellow arrows point to distal side, and white arrows point to proximal side. The presence of E-cad staining confirms that low or absent Dachs staining on the proximal side is not simply due to a difference in focal plane.
(6.06 MB TIF)

Figure S5 Additional studies of Zyx localization in wing imaginal discs. (A–D) show close-ups of wing imaginal discs, stained for E-cad (blue) and Zyx:V5 (red), with MARCM clones expressing Zyx:V5, and (A) wild-type control, (B) *dachs^GC13^* mutant, (C) *fat^8^* mutant, and (D) *ex^e1^* mutant. (E–F) Horizontal (E) and vertical (F) sections through a wing disc stained for Fat (red), with posterior cells marked by GFP (blue), and with *en-Gal4 UAS-dcr2 UAS-GFP UAS-RNAi-Zyx^32018^* transgenes. (G–N) Horizontal (G,I,K,M) and vertical (H,J,L,N) sections through a wing disc stained for Ex (red), with posterior cells marked by GFP (blue), and with *en-Gal4 UAS-dcr2 UAS-GFP* and (G,H) no additional transgenes (control), (I,J) *UAS-RNAi-Zyx^32018^*, (K,L) *UAS-RNAi-fat*, or (M,N) *UAS-RNAi-fat UAS-RNAi-Zyx^32018^* transgenes. (O,P) Horizontal (M) and vertical (N) sections through a wing disc stained for F-actin (using phalloidin, yellow), with posterior cells marked by GFP (blue), and with *en-Gal4 UAS-dcr2 UAS-GFP UAS-RNAi-Zyx^32018^* transgenes.
(9.83 MB TIF)

Figure S6 Additional studies of binding amongst Zyx, Jub, Dachs, and Wts. Western blots on co-immunoprecipitation experiments, with upper two blots indicating the relative amount of protein in the lysates used for the experiments, and the lower panel indicating the material co-precipitated by the indicated antibody. GFP serves as a negative control. (A) Co-precipitation of V5-tagged Dachs and Zyx-LD with the FLAG-tagged Wts or GFP control, as indicated at top. Addition of Dachs:V5 (3x refers to amounts used in Figure 6G) does not increase precipitation of Zyx-LD with Wts. Arrows identify the indicated proteins. (B) Co-precipitation of V5-tagged Dachs with the FLAG-tagged proteins indicated at top. The results show that Dachs binds to Zyx much more strongly than it does to Jub.
(1.22 MB TIF)

Figure S7 Characterization of the influence of *Jub* on Yki activity. All panels show *en-Gal4 UAS-dcr2 UAS-RNAi-Jub^38442^* third instar wing imaginal discs. (A) Stained for *th-lacZ* (red), with posterior cells marked by Dcr2 (blue). (B) Stained for Diap1 (red) and *ex-lacZ* (green), with posterior cells marked by GFP (blue). (C,D) Stained for Yki (red/white) and nuclei (based on nuclear localization of ß-galactosidase, green/white) with posterior cells marked by GFP (blue) or demarcated by the dashed line. In (C), upper panels show a horizontal section, and lower panels show a vertical section; (D) shows a close-up of a portion of the image shown in (C).
(5.85 MB TIF)

Table S1 Primary screening of RNAi lines. All fly lines for the primary screening were obtained from the NIG collection. The first two columns identify the RNAi line and the gene (some genes are represented by two independent RNAi lines). The genes screened included all of the lines targeted against X chromosome genes that were available at the time the screen was initiated, plus a selection of lines for 4^th^ chromosome genes, kinases, phosphatases, and myosins. The third column indicates the phenotype when RNAi lines were crossed to a *pnr-Gal4 UAS-dcr2* chromosome. Pnr is expressed in a broad stripe along the center of the notum. A blank entry means that no visible phenotype was detected. The fourth column indicates the phenotype when RNAi lines were crossed to a *vg-Gal4 UAS-dcr2* chromosome. Vg is expressed in a broad stripe along the dorsal-ventral compartment boundary, mostly in the wing but also extending into the hinge and notum tissue. A blank entry means that no visible phenotype was detected. The fifth column indicates the phenotype when RNAi lines were crossed to a *ci-Gal4 UAS-dcr2* chromosome. Ci is expressed in anterior cells. For this cross, we only examined third instar wing imaginal discs, which were stained with antibodies against Diap1 and Wg. For this column, a blank entry means that this genotype was not examined.
(0.15 MB XLS)

Text S1 Supplementary methods.
(0.05 MB DOC)

## Abbreviations

PCP: planar cell polarity
