# Supplementary figures and images for: Zyxin Links Fat Signaling to the Hippo Pathway
Source: PLoS Biol. 2011 Jun 7;9(6):e1000624. doi: 10.1371/journal.pbio.1000624 (PMC3110180; doi:10.1371/journal.pbio.1000624)

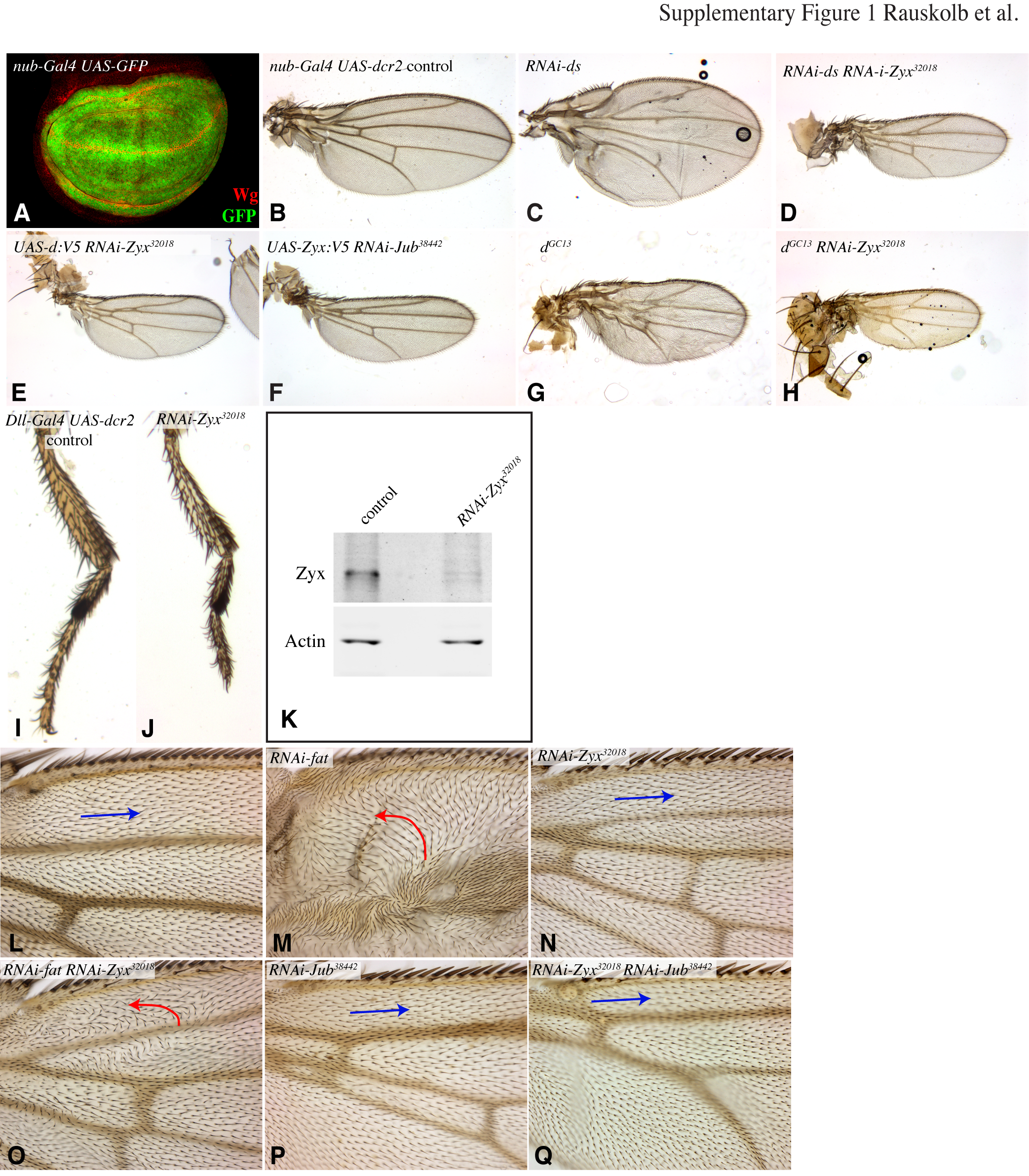

Supplement: Figure S1 — Additional characterization of the influence of Zyx and Jub on wing and leg growth and PCP. (A) Wing imaginal disc from nub-Gal4 UAS-dcr2 UAS-GFP larva; the nub expression domain is indicated by GFP expression (green); for reference Wg expression (red) is also shown. Panels (B–F) show wings from male adults flies with nub-Gal4 UAS-dcr2, and (B) no additional transgenes (control), (C) UAS-RNAi-ds, (D) UAS-RNAi-ds UAS-RNAi-Zyx32018, (E) UAS-dachs:V5 UAS-RNAi-Zyx32018, and (F) UAS-Zyx:V5 UAS-RNAi-Jub38442. Panels (G,H) show wings from male adults flies of (G) dGC13 nub-Gal4 and (H) dGC13 nub-Gal4 UAS-RNAi-Zyx32018. (I) Leg from Dll-Gal4 UAS-dcr2 adult male control. (J) Leg from Dll-Gal4 UAS-dcr2 UAS-RNAi-Zyx32018 adult male. (K) Western blot on lysates of third instar wing discs from tub-Gal4 UAS-dcr2 (control) and tub-Gal4 UAS-dcr2 UAS-RNAi-Zyx32018 (RNAi-Zyx32018) probed with anti-Zyx and anti-Actin antisera, as indicated. Similar amounts of total protein were loaded in each lane. (L–Q) show close-ups of the anterior wing from male adults flies with nub-Gal4 UAS-dcr2, and (L) no additional transgenes (control), (M) UAS-RNAi-fat, (N) UAS-RNAi-Zyx32018, (O) UAS-RNAi-fat UAS-RNAi-Zyx32018, (P) UAS-RNAi-Jub38442, and (Q) UAS-RNAi-Zyx32018 UAS-RNAi-Jub38442. Blue arrows indicate normal polarity; red arrows indicate disturbed polarity. (8.60 MB TIF) [file pbio.1000624.s001.tif]

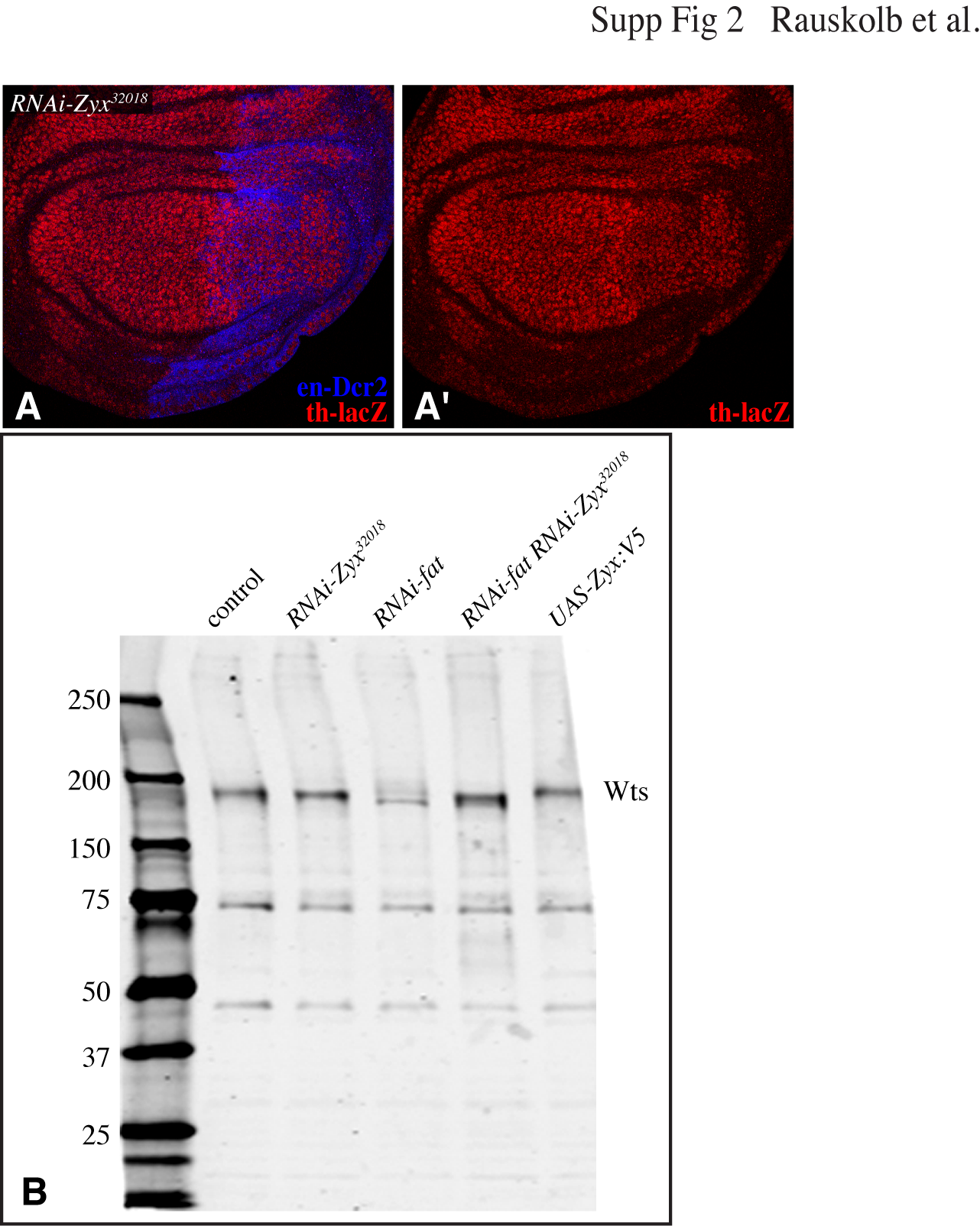

Supplement: Figure S2 — Additional characterization of the influence of Zyx on Yki activity. (A) Third instar en-Gal4 UAS-dcr2 UAS-RNAi-Zyx32018 wing imaginal disc, stained for th-lacZ (red), with posterior cells marked by Dcr2 (blue). (B) Western blot on lysates of third instar wing discs from tub-Gal4 UAS-dcr2 control (+), tub-Gal4 UAS-dcr2 UAS-RNAi-Zyx32018, tub-Gal4 UAS-dcr2 UAS-RNAi-fat, tub-Gal4 UAS-dcr2 UAS-RNAi-fat UAS-RNAi-Zyx32018, and UAS-Zyx:V5, probed with anti-Wts. This panel shows the entire blot for the bands depicted in Figure 5A. The Wts band was identified based on its mobility and the observation that this band is decreased by wts RNAi. Numbers indicate the calculated mobilities of the size markers. (1.46 MB TIF) [file pbio.1000624.s002.tif]

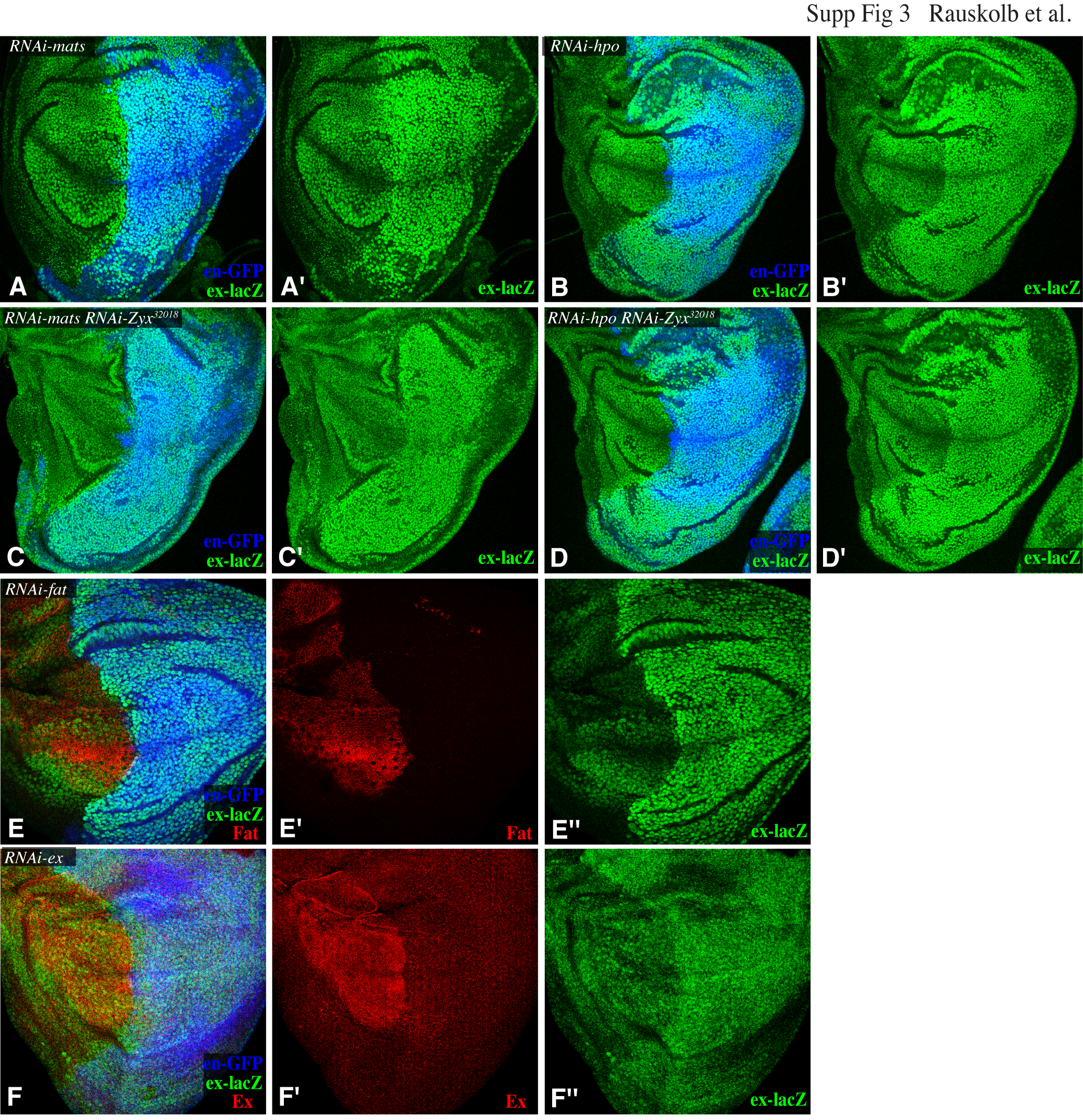

Supplement: Figure S3 — Additional characterization of the epistatic relationship of Zyx to the Hippo pathway. Wing imaginal discs, stained for ex-lacZ (green), with posterior cells marked by GFP (blue), and with en-Gal4 UAS-dcr2 UAS-GFP transgenes, and (A) UAS-RNAi-mats, (B) UAS-RNAi-hpo, (C) UAS-RNAi-mats UAS-RNAi-Zyx32018, (D) UAS-RNAi-hpo UAS-RNAi-Zyx32018, (E) UAS-RNAi-fat, and (F) UAS-RNAi-ex. Discs in (E and F) are also stained for anti-Fat (red, E) and anti-Ex (red, F). Both RNAi lines are highly effective, but the anti-Ex sera gives higher background staining. (7.74 MB TIF) [file pbio.1000624.s003.tif]

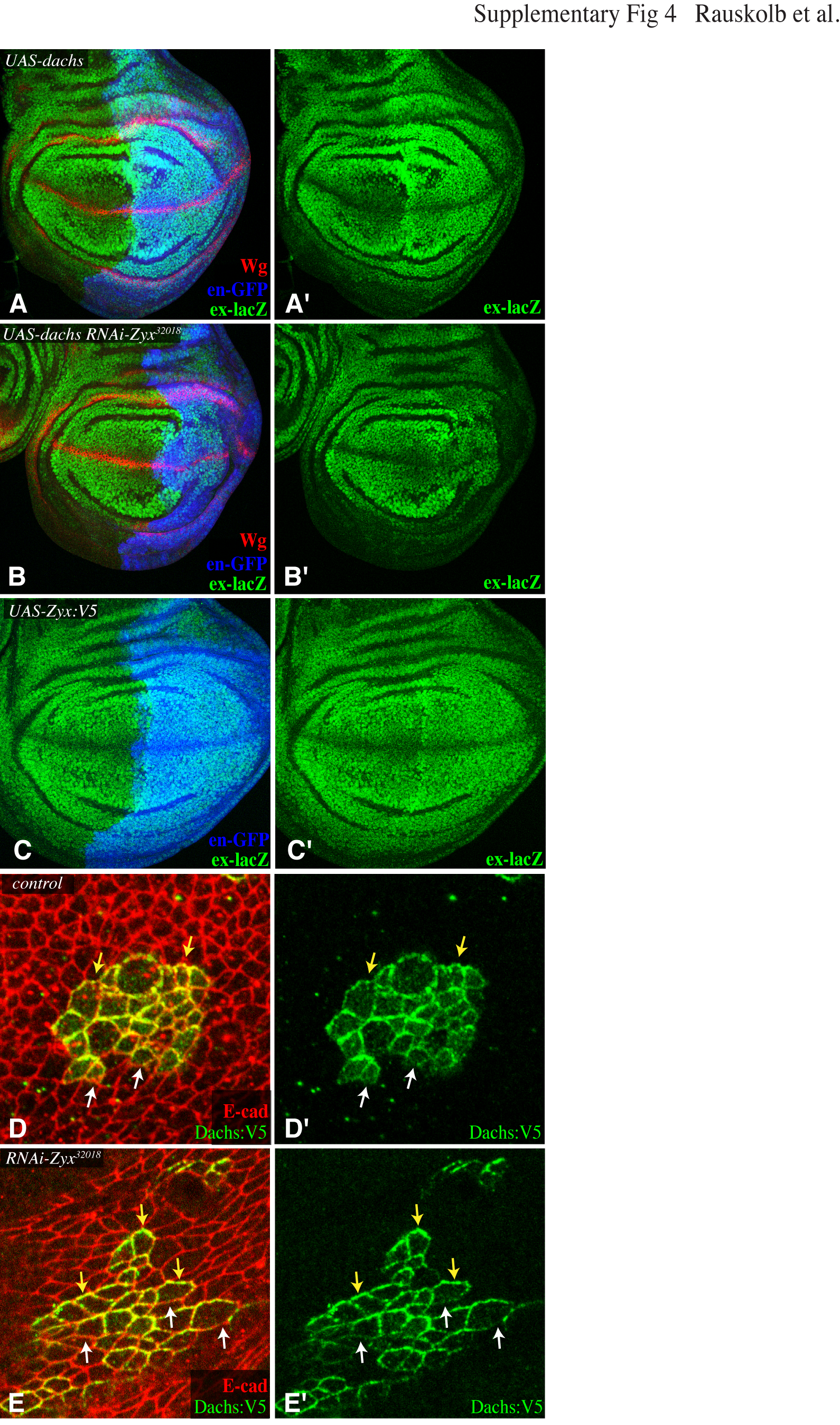

Supplement: Figure S4 — Additional studies of Zyx epistasis and Zyx localization in wing imaginal discs. (A,B) Wing imaginal discs, stained for Wg (red) and ex-lacZ (green), with posterior cells marked by GFP (blue), and with en-Gal4 UAS-dcr2 UAS-GFP transgenes, and (A) UAS-dachs:V5 or (B) UAS-dachs:V5 UAS-RNAi-Zyx32018 transgenes. (C) en-Gal4 UAS-dcr2 UAS-GFP UAS-Zyx:V5 wing imaginal disc, stained for ex-lacZ (green), with posterior cells marked by GFP (blue). (D,E) Close-ups of wing imaginal discs, stained for E-cad (red), showing clones of cells expressing Dachs:V5 (green), under AyGal4 control, with AyGal4 UAS-dcr2 UAS-dachs:V5 transgenes, and (D) no additional transgenes (control) or (E) UAS-RNAi-Zyx32018. Yellow arrows point to distal side, and white arrows point to proximal side. The presence of E-cad staining confirms that low or absent Dachs staining on the proximal side is not simply due to a difference in focal plane. (6.06 MB TIF) [file pbio.1000624.s004.tif]

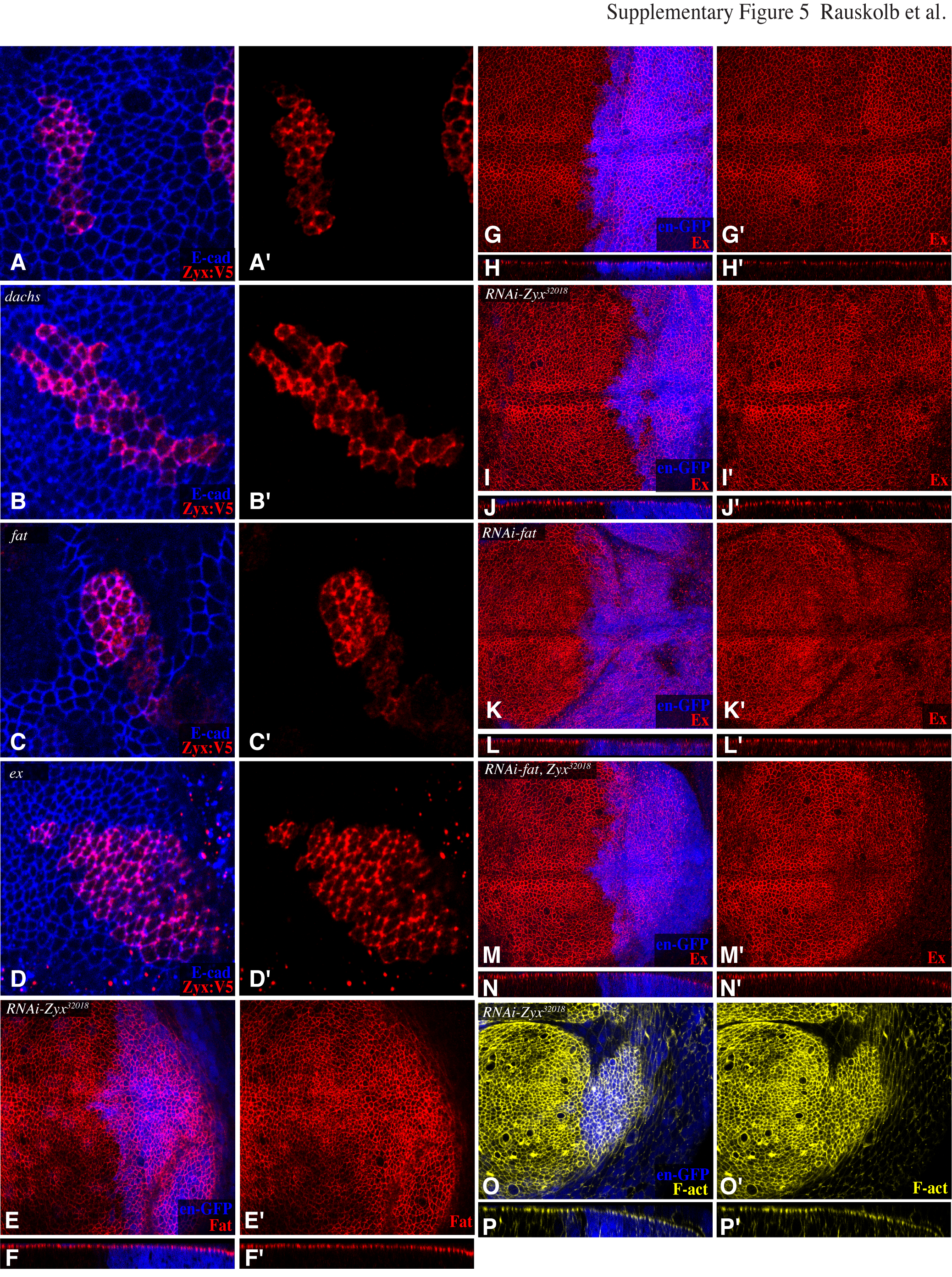

Supplement: Figure S5 — Additional studies of Zyx localization in wing imaginal discs. (A–D) show close-ups of wing imaginal discs, stained for E-cad (blue) and Zyx:V5 (red), with MARCM clones expressing Zyx:V5, and (A) wild-type control, (B) dachsGC13 mutant, (C) fat8 mutant, and (D) exe1 mutant. (E–F) Horizontal (E) and vertical (F) sections through a wing disc stained for Fat (red), with posterior cells marked by GFP (blue), and with en-Gal4 UAS-dcr2 UAS-GFP UAS-RNAi-Zyx32018 transgenes. (G–N) Horizontal (G,I,K,M) and vertical (H,J,L,N) sections through a wing disc stained for Ex (red), with posterior cells marked by GFP (blue), and with en-Gal4 UAS-dcr2 UAS-GFP and (G,H) no additional transgenes (control), (I,J) UAS-RNAi-Zyx32018, (K,L) UAS-RNAi-fat, or (M,N) UAS-RNAi-fat UAS-RNAi-Zyx32018 transgenes. (O,P) Horizontal (M) and vertical (N) sections through a wing disc stained for F-actin (using phalloidin, yellow), with posterior cells marked by GFP (blue), and with en-Gal4 UAS-dcr2 UAS-GFP UAS-RNAi-Zyx32018 transgenes. (9.83 MB TIF) [file pbio.1000624.s005.tif]

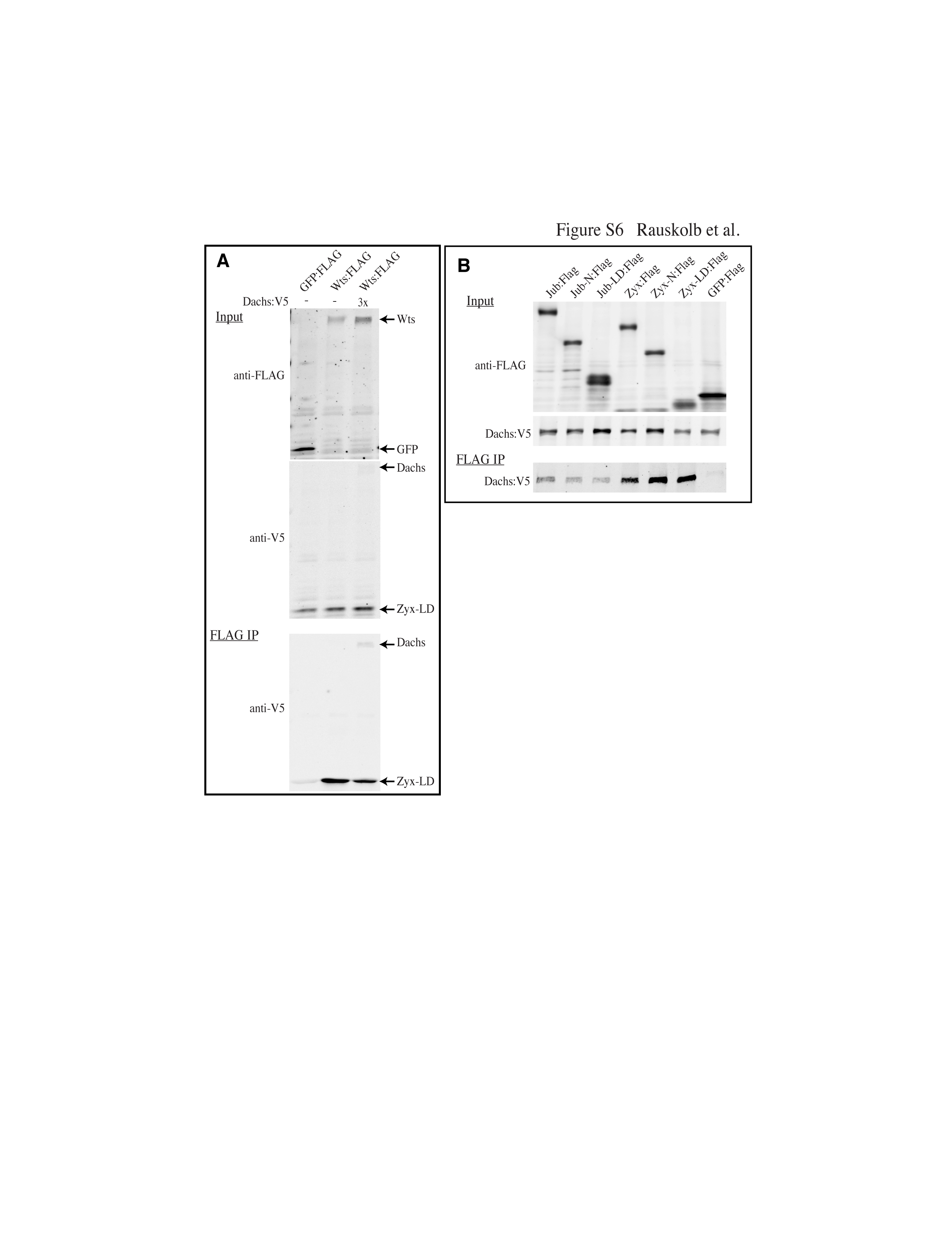

Supplement: Figure S6 — Additional studies of binding amongst Zyx, Jub, Dachs, and Wts. Western blots on co-immunoprecipitation experiments, with upper two blots indicating the relative amount of protein in the lysates used for the experiments, and the lower panel indicating the material co-precipitated by the indicated antibody. GFP serves as a negative control. (A) Co-precipitation of V5-tagged Dachs and Zyx-LD with the FLAG-tagged Wts or GFP control, as indicated at top. Addition of Dachs:V5 (3x refers to amounts used in Figure 6G) does not increase precipitation of Zyx-LD with Wts. Arrows identify the indicated proteins. (B) Co-precipitation of V5-tagged Dachs with the FLAG-tagged proteins indicated at top. The results show that Dachs binds to Zyx much more strongly than it does to Jub. (1.22 MB TIF) [file pbio.1000624.s006.tif]

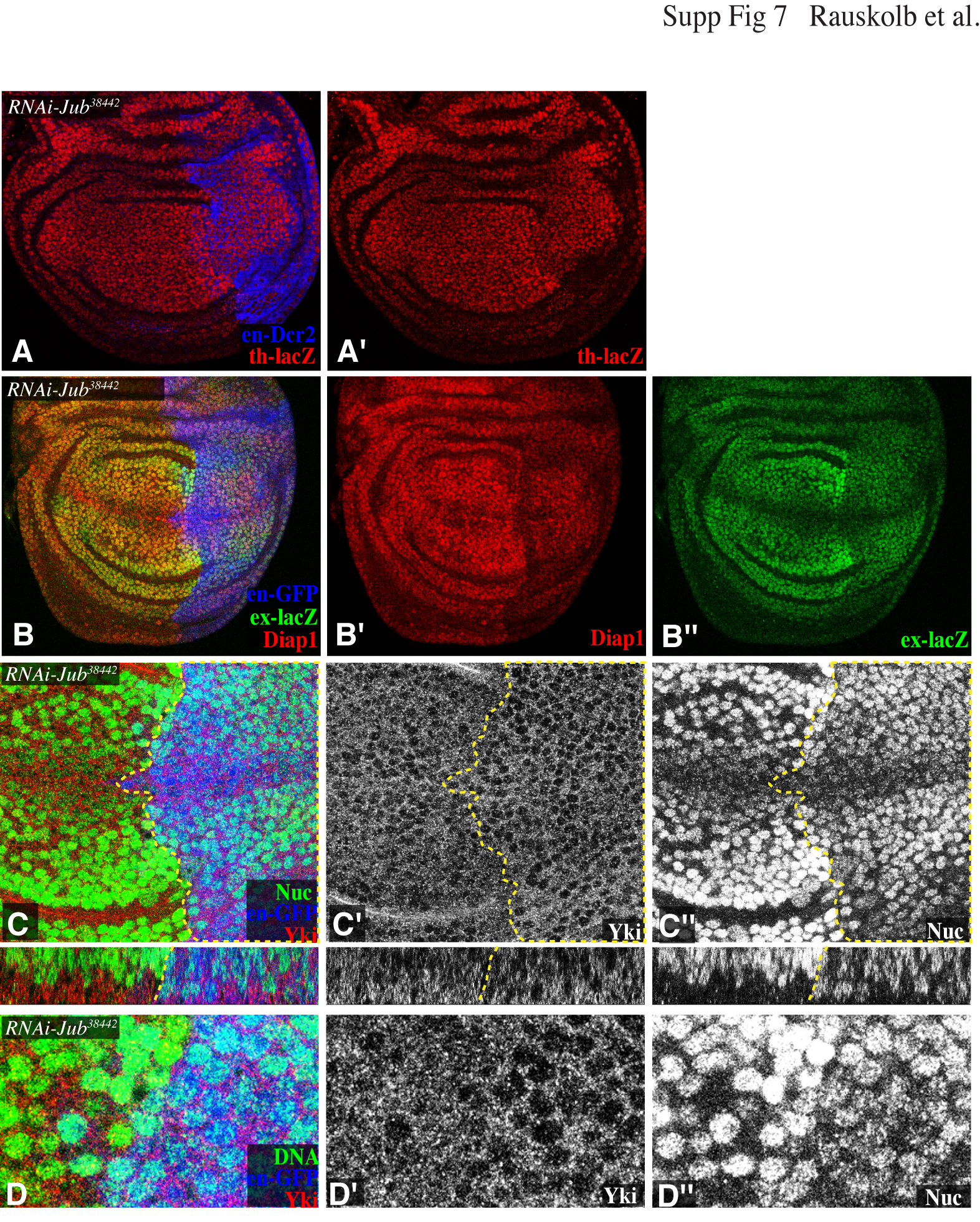

Supplement: Figure S7 — Characterization of the influence of Jub on Yki activity. All panels show en-Gal4 UAS-dcr2 UAS-RNAi-Jub38442 third instar wing imaginal discs. (A) Stained for th-lacZ (red), with posterior cells marked by Dcr2 (blue). (B) Stained for Diap1 (red) and ex-lacZ (green), with posterior cells marked by GFP (blue). (C,D) Stained for Yki (red/white) and nuclei (based on nuclear localization of ß-galactosidase, green/white) with posterior cells marked by GFP (blue) or demarcated by the dashed line. In (C), upper panels show a horizontal section, and lower panels show a vertical section; (D) shows a close-up of a portion of the image shown in (C). (5.85 MB TIF) [file pbio.1000624.s007.tif]
