# Supplementary material for: Zyxin Links Fat Signaling to the Hippo Pathway
Source: PLoS Biol. 2011 Jun 7;9(6):e1000624. doi: 10.1371/journal.pbio.1000624 (PMC3110180; doi:10.1371/journal.pbio.1000624)
Supplement: Text S1 — Supplementary methods. (0.05 MB DOC) [file pbio.1000624.s009.doc]

**Text S1**

**Supplementary methods.**

**Plasmid Constructs and Transfections**

**UAS-Zyx:V5**: A full length Zyx cDNA (2021bp, including 5'UTR, coding sequence from nt 181 to nt 1938, and 3'UTR) from clone *zyx102.44* (plasmid pEXlox44a) was amplified by PCR (using primers 5'- AATTCGTTAACAGATCTGCGGCCGCATAAATTCGATAAGCGATAG-3' and 5'- CCTCTAGAGGTACCCTCGAGTTTTTTTATTATTATCAATTTATTTTCCGA-3'), and cloned into NotI/XhoI digested pUAST-attB. A V5-epitope-tag, plus poly-glycine linker, was added (using primers 5'-CCGCATGACGTCAGAACATGGAGGAGGAGGAGGTAAGCCTATCCCTAA- 3' and 5' –TAAAATGAGCACTCAATTTAACCGGTACGCGTAGAATCGAGA-3'), C-terminal to the last amino acid of Zyx using the QuikChange Site-Directed Mutagenesis Kit (Stratagene) to make pUAS-Zyxin:V5. This plasmid was transformed into an attP site at 68A (attP2) to make UAS-Zyx:V5 flies.

**UAS-Zyx-LD:V5**: nt 1336 to nt 2021 of Zyx (aa 386-585, plus 3' UTR and V5 tag) was amplified by PCR using UAS-Zyxin:V5 as template, plus addition of an ATG, using the forward primer 5'- TAACAGATCTGCGGCCGCATGGGTAGGTGTGTCAAATGCA-3' and the reverse primer 5'-CCTCTAGAGGTACCCTCGAGTTTTTTTATTATTATCAATTTATTTTCCGA-3', and then cloned into NotI/XhoI digested pUAST-attB to make UAS-Zyx-LD:V5.

**UAS-FLAG:Zyx** constructs: Full length Zyx (nt 184-nt 2021, encoding aa 2-585 plus 3'UTR), Zyx-LD (nt 1336 to nt 2021, encoding aa 386-585 plus 3'UTR), and Zyx-N (nt 184 to nt 1335, encoding aa 2-385) were amplified by PCR from pEXlox44a using forward primers: 5'- CGCGGTACCGAGTCTGTGGCCCAGCAACTTA-3', or 5'- CGCGGTACCGGTAGGTGTGTCAAATGCA-3', and reverse primers: 5'- TGCACTAGTTTTTTTATTATTATCAATTTATTTTCCGA-3', or 5'- TGCACTAGTTTAGTAGTTTTCCAATTCTTGAACC -3'), and cloned into KpnI/XbaI digested pUAST-Flag:Lft , replacing the *lft* gene of this plasmid.

**UAS-FLAG:Jub** constructs: An N-terminal triple FLAG tag was added to Full length Jub (aa 1-728), Jub-LD (aa 467-728), and Jub-N (aa 1-466 by PCR from pUAST-mCherry-djub, (using forward primers: 5’-CGCGGTACCATGACCACCCAGCGGACGCAGAC-3’, or 5’- CGCGGTACCGGTCTCACCAAGAATCTGCTA-3’, and reverse primers: 5’-TGCACTAGTTTATCTCCTGGGTTTCAAGGCA-3’, or 5’-TGCACTAGTTTATCCCATATACTGGTACGAA-3’), then cloning into KpnI/XbaI digested pUAST-Flag:Lft, replacing the *lft* gene of this plasmid.

**FLAG:Wts constructs:** Full length Wts (pUAST-FLAG:Wts), N-terminal 708aa (pUAST- FLAG:Wts∆C398), N-terminal 192aa (pUAST-FLAG:Wts∆C914), C-terminal 914aa (pUAST- FLAG:Wts∆N192), and C-terminal 398aa (pUAST-FLAG:Wts∆N708) were amplified by PCR from pUAST-wts:Myc , and cloned into KpnI/XbaI digested pUAST-Flag:Lft , replacing the *lft* gene of this plasmid.

**UAS-Jub:V5** was constructed by combining RE22016, which contains the 5' end of *Jub*, and RE33016, which contains the 3' end of *Jub*. The resulting construct encodes a protein corresponding to Jub-PC (Flybase), and lacks the putative second exon (AVNAALKPRR) of Jub-PB. A V5-epitope-tag was added to the C-terminus using the In-Fusion Advantage PCR Cloning Kit (Clontech), and Jub:V5 was then cloned into pUAST-attB to make pUAS-Jub:V5. UAS-Jub-LD:V5 contains sequences starting from the second exon of Jub-PC, 49 aa N-terminal to the first LIM domain.

**pUAST-dachs:V5** has been described previously ^6^. pUAST-*dachs*-M:V5 (myosin domain) includes nucleotides 867-3216 of GenBank CG10595-PA, and was created by PCR amplifying, with addition of an ATG, and inserting into EcoRI/XbaI digested pUAST-*dachs*:V5 to replace the full length *dachs*.

All plasmid constructs were verified by DNA sequencing.

**Transfections**

Transfections into S2 cells were performed essentially as described previously . Where possible amounts of DNA transfected were optimized to obtain similar expression levels of related proteins. Amounts of DNA used in standard transfections (with 2 x 10^6^ cells, in 6 well plates) were:

pUAS-Zyx:V5: 500 ng, except for experiments examining stimulation of Wts binding by Dachs, when only 250 ng was used to compensate for the lower experession of Dachs:V5.

UAS-Zyx-LD:V5: 1500-2000 ng

UAS-Flag:Zyx: 500 ng

UAS-Flag:Zyx-N: 500 ng

UAS-Flag:Zyx-LD: 1500-2000 ng

pUAST-FLAG:Wts: 400 ng

pUAST-FLAG:Wts∆C398: 550 ng

pUAST-FLAG:Wts∆C914: 220 ng

pUAST-FLAG:Wts∆N192: 400 ng

pUAST-FLAG:Wts∆N708: 1300 ng

pUAST-Dachs:V5 1500-2000 ng, except for experiments examining stimulation of Wts binding by Dachs, when only 250 ng (=1x), 750 ng (=3x) or 1500 ng (=6x) was used.

pUAST-Dachs-M:V5: 500 ng

UAS- FLAG:Jub: 250 ng

UAS- FLAG:Jub-N: 375 ng

UAS- FLAG:Jub-LD 500 ng
